# Acetylation of Surface Carbohydrates in Bacterial Pathogens Requires Coordinated Action of a Two-Domain Membrane-Bound Acyltransferase

**DOI:** 10.1128/mBio.01364-20

**Published:** 2020-08-25

**Authors:** Caroline R. Pearson, Sarah N. Tindall, Reyme Herman, Huw T. Jenkins, Alex Bateman, Gavin H. Thomas, Jennifer R. Potts, Marjan W. Van der Woude

**Affiliations:** a York Biomedical Research Institute, University of York, York, United Kingdom; b Department of Biology, University of York, York, United Kingdom; c Hull York Medical School, University of York, York, United Kingdom; d York Structural Biology Laboratory, Department of Chemistry, University of York, York, United Kingdom; e European Molecular Biology Laboratory, European Bioinformatics Institute (EMBL-EBI), Wellcome Genome Campus, Hinxton, Cambridgeshire, United Kingdom; University of Guelph; Harvard Medical School

**Keywords:** O-antigen, SGNH superfamily, *Salmonella*, acetylation, acyltransferase-3 family, Gram-negative bacteria, lipopolysaccharide, membrane proteins, structure-activity relationship, surface antigens

## Abstract

Membrane bound acyltransferase-3 (AT3) domain-containing proteins are implicated in a wide range of carbohydrate O-acyl modifications, but their mechanism of action is largely unknown. O-antigen acetylation by AT3 domain-containing acetyltransferases of *Salmonella* spp. can generate a specific immune response upon infection and can influence bacteriophage interactions. This study integrates *in situ* and *in vitro* functional analyses of two of these proteins, OafA and OafB (formerly F2GtrC), which display an “AT3-SGNH fused” domain architecture, where an integral membrane AT3 domain is fused to an extracytoplasmic SGNH domain. An *in silico*-inspired mutagenesis approach of the AT3 domain identified seven residues which are fundamental for the mechanism of action of OafA, with a particularly conserved motif in TMH1 indicating a potential acyl donor interaction site. Genetic and *in vitro* evidence demonstrate that the SGNH domain is both necessary and sufficient for lipopolysaccharide acetylation. The structure of the periplasmic SGNH domain of OafB identified features not previously reported for SGNH proteins. In particular, the periplasmic portion of the interdomain linking region is structured. Significantly, this region constrains acceptor substrate specificity, apparently by limiting access to the active site. Coevolution analysis of the two domains suggests possible interdomain interactions. Combining these data, we propose a refined model of the AT3-SGNH proteins, with structurally constrained orientations of the two domains. These findings enhance our understanding of how cells can transfer acyl groups from the cytoplasm to specific extracellular carbohydrates.

## Abstract (precis)

Acyltransferase-3 (AT3) domain-containing membrane proteins are involved in *O*-acetylation of a diverse range of carbohydrates across all domains of life. In bacteria they are essential in processes including symbiosis, resistance to antimicrobials, and biosynthesis of antibiotics. Their mechanism of action, however, is poorly characterized. We analyzed two acetyltransferases as models for this important family of membrane proteins, which modify carbohydrates on the surface of the pathogen Salmonella enterica, affecting immunogenicity, virulence, and bacteriophage resistance. We show that when these AT3 domains are fused to a periplasmic partner domain, both domains are required for substrate acetylation. The data show conserved elements in the AT3 domain and unique structural features of the periplasmic domain. Our data provide a working model to probe the mechanism and function of the diverse and important members of the widespread AT3 protein family, which are required for biologically significant modifications of cell-surface carbohydrates.

## INTRODUCTION

*Salmonella* infections are a considerable public health burden in both developing and developed countries. Salmonella enterica subspecies *enterica* serovar Typhimurium is estimated to cause more than 150,000 human deaths from gastroenteritis each year (1, 2). A sublineage of this serovar is the dominant cause of invasive nontyphoidal *Salmonella* (iNTS) bloodstream infections in Africa (3). The Typhi serovar of this subspecies is the major cause of typhoid fever, resulting in over 200,000 deaths annually (2, 4). In the United States, there are over 10,000 cases annually of these serovars combined (5, 6).

Cell surface lipopolysaccharide (LPS) is an important virulence factor. The O-antigen, the most distal and variable portion of LPS, is composed of repeating oligosaccharide units whose composition and structure vary between species and, in the case of *Salmonella* spp., between serovars. Modification of the O-antigen by alteration of sugar linkages or addition of moieties such as glucose or acetate (7, 8) can influence immunogenicity and virulence, and confer resistance to lytic phage infection (9–12).

Carbohydrates on the bacterial cell surface are frequently *O*-acetylated by acyltransferase proteins which contain a 10-transmembrane helix (TMH) acyltransferase-3 (AT3) (InterPro IPR002656 and Pfam PF01757; also known as acyltransferase_3/putative acetyl-CoA transporter, Transporter Classification Database number [TC] number 9.B.97). This family of proteins is widespread in eukaryotes and prokaryotes and is involved in a range of acylation modifications. Examples of AT3-containing acetyltransferases from prokaryotes include those mediating peptidoglycan acetylation and contributing to lysozyme resistance (13, 14), modification of root nodulation factors to initiate symbioses (15), and O-antigen acetylation (9, 16, 17). Despite the involvement of AT3-containing proteins in a wide range of reactions, their mechanism and structure are poorly characterized.

Among bacterial AT3 carbohydrate acetyltransferases, there are two known domain architectures, proteins consisting of an AT3 domain only (AT3-only) and an N-terminal AT3 domain linked to an extracytoplasmic domain, commonly an SGNH domain (AT3-SGNH fused). The SGNH domain is fused through addition of an 11th TMH and linking region. Oac (in *Shigella* spp.) is an example of an AT3-only protein that is essential for O-antigen acetylation (18), whereas OatA, the *O*-acetyltransferase of peptidoglycan in *Staphylococcus* spp., is an example of an AT3-SGNH fused protein (14). SGNH domains (InterPro number IPR036514) are a large and diverse family of small catalytic domains of around 200 amino acids, originally characterized as a subgroup of the GDSL hydrolase family based on their invariant residues, Ser, Gly, Asn, His—hence, SGNH—which occur in four blocks of conserved sequence (19, 20). Members of this family that are active against carbohydrates are also classified as CE3 family proteins in the carbohydrate-active enzymes database (CAZy) (21). Subsequently, many more proteins have been found to belong to this diverse family, and they no longer fully adhere to the original paradigm of SGNH. However, most members typically contain a catalytic triad of Ser, His, and Asp and oxyanion hole residues within the four blocks of conserved sequence (22). It is not clear how the AT3 and SGNH domains function together in AT3-SGNH fused carbohydrate acetyltransferases, nor how the AT3-only proteins function independently of a linked periplasmic domain.

In *Salmonella* spp., there are two defined O-antigen acetyltransferases, OafA and OafB (9, 10, 17, 23). Slauch et al. determined that the integral membrane protein OafA from *S.* Typhimurium (17) acetylates the 2-hydroxyl group on the abequose moiety of the O-antigen unique to this serovar (24). This results in acquisition of the O:5 serotype (defined by the Kauffmann-White-Le Minor scheme) (25, 26), which is required for production of protective antibodies against *S.* Typhimurium infection (24, 27). Multiple *Salmonella* serovars have a rhamnose moiety in the O-antigen that can be acetylated at the 2- and 3-hydroxyl groups by F2GtrC proteins (9, 10, 23). As it is clear that F2GtrC is an acetyltransferase with no functional relationship to the GtrABC glycosylating proteins, we propose to rename this and orthologous rhamnose acetyltransferases OafB. The name reflects the protein architecture (O-antigen acetyltransferase-fused B), similar to what we suggest for OafA (O-antigen acetyltransferase-fused A).

In this work, using *in situ* and *in vitro* functional analysis of OafA and OafB O-antigen acetyltransferases, we address the following key questions to further our understanding of the mechanism of acetyl transport and transfer in AT3-SGNH fused acetyltransferases. (i) Are there essential residues in the membrane-bound AT3 domain that can give clues to their role in acetyl transfer? (ii) Can we obtain insight into the architecture of these proteins by elucidating the structure of the SGNH domain and its N-terminal extension? (iii) What is the function of the SGNH domain, and can it function independently of the AT3 domain?

## RESULTS

### *In silico* analysis identifies conserved features in the integral membrane domains of bacterial AT3 acetyltransferases.

**FIG 1:**
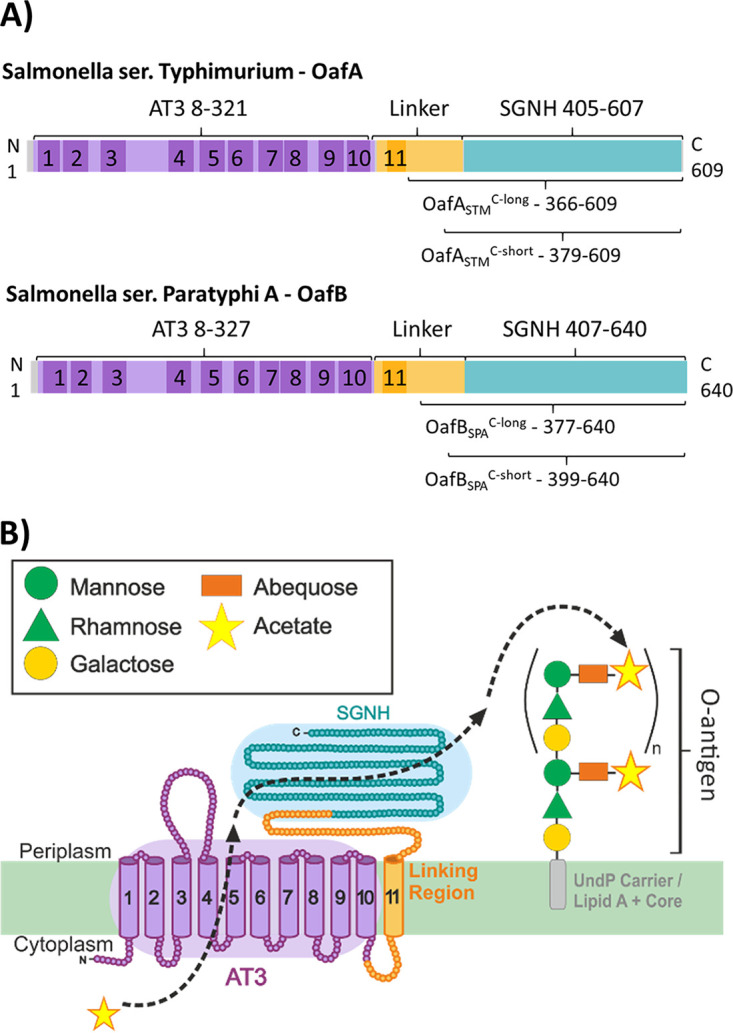
OafA and OafB are membrane-bound *O*-acetyltransferases that acetylate the O-antigen of *Salmonella*. (A) Schematic representation of OafA and OafB functional (colored) and transmembrane (shaded) domains predicted by InterPro and TMHMM, respectively. C-term constructs used for *in vitro* characterization are indicated below the protein. (B) Proposed mechanism of action of O-antigen acetyltransferases during maturation of the LPS in the periplasm using OafA as an example. AT3, InterPro IPR002656; SGNH, InterPro IPR013830.

The *S.* Typhimurium O-antigen acetyltransferases OafA (17) and OafB (23) (formerly F2GtrC) are both predicted by InterPro to contain an N-terminal AT3 domain (InterPro IPR002656, Pfam PF01757) fused to an SGNH domain (InterPro IPR013830, Pfam PF14606, or Pfam PF13472) (28, 29) (Fig. 1A). The AT3 domain has 10 TMH and an additional 11th helix that is presumably required to localize the fused SGNH domain in the periplasm (Fig. 1A) (30); this prediction is supported by experimental topology analysis of OafB (9) and consistent with topology analysis of Oac (31), a comparison enabled by our detailed alignments (see below). Reinforcing the widespread functions of these understudied proteins in bacteria, we identified in the literature 30 bacterial AT3 domain-containing proteins with experimentally confirmed carbohydrate acetyltransferase activity (9, 14–17, 32–55). Of these 30 proteins, 19 contain just the AT3 domain, while 11, including OafA and OafB, have the fused AT3-SGNH architecture (see Table S1 in the supplemental material). Previous work showed that in OafA and OafB, the SGNH domain is essential for acetyltransferase activity (9, 56), and thus, we propose the following working model for the mechanism of action (Fig. 1B). In AT3-SGNH proteins, the AT3 domain passes an acetyl group from an unidentified donor in the cytoplasm to the periplasmic face of the inner membrane. This acetyl group is then transferred to the SGNH domain, which catalyzes specific carbohydrate *O*-acetylation (Fig. 1B). To test this model, we first determined whether residues conserved between AT3-only and AT3-SGNH acetyltransferases are important for acetyltransferase activity.

10.1128/mBio.01364-20.8 TABLE S1 Experimentally characterized bacterial AT3 acetyltransferases. Download Table S1, PDF file, 0.4 MB. Copyright © 2020 Pearson et al. 2020 Pearson et al. This content is distributed under the terms of the Creative Commons Attribution 4.0 International license.

**FIG 2:**
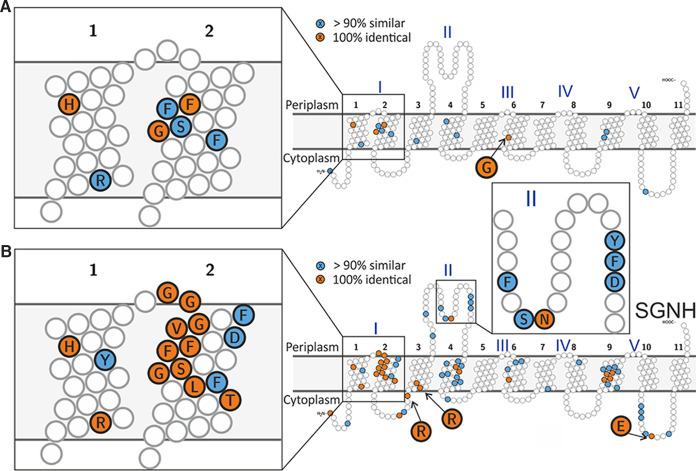
Conservation in transmembrane domains of experimentally characterized bacterial AT3 carbohydrate acetyltransferases. The 100% identical residues are colored orange, similar residues in >90% sequences are colored blue, and conserved small hydrophobic residues in transmembrane helices were not colored. (A) Conserved residues across all 30 currently known experimentally characterized proteins and OafB-SPA. (B) Conservation in only AT3-SGNH fused proteins in the alignment. See Table S1 for details of aligned sequences and Fig. S1 for full alignment.

Alignments of the 30 characterized AT3 acetyltransferases along with an S. enterica serovar Paratyphi A OafB homologue revealed that only 4 amino acids are invariant across all 31 proteins, OafA_H25_, OafA_F41,_ OafA_G46_, and OafA_G202_ (Fig. 2A, Fig. S1). OafA_F41_ and OafA_G46_ belong to the FFXISG motif previously identified in unfused AT3 O-antigen acetyltransferases (Fig. S1) (31). Two conserved residues are predicted in TMH1, separated by 10 amino acids, in an R/K-X_10_-H motif (Fig. 2A, Fig. S1). A previously identified RXXR motif (OafA_R69,R72_) in loop 2-3 is essential for activity in Shigella flexneri Oac (Oac_R73,R75_) (57) and OafB (OafB_R71,R73_) (9). This motif is highly (but not absolutely) conserved across the 31 analyzed acetyltransferases.

10.1128/mBio.01364-20.1 FIG S1 Alignment of characterized AT3 acetyltransferases. Protein sequences are in the same order as in Table S1 after Salmonella enterica serovar Paratyphi A OafB WP_00400612. SGNH fused acetyltransferases are indicated by a gray box. Asterisks mark residues selected for mutation from this alignment. Download FIG S1, PDF file, 1.8 MB. Copyright © 2020 Pearson et al. 2020 Pearson et al. This content is distributed under the terms of the Creative Commons Attribution 4.0 International license.

We next examined features unique to the AT3 domains of AT3-SGNH fused acetyltransferases; these 11 sequences derive from diverse Gram-positive and Gram-negative bacteria (Fig. 2B, Table S1). The most striking shared feature of AT3-SGNH fused proteins is the highly conserved GG-F/Y-XGV-D/P/V motif located at the periplasmic side of TMH2 (OafA_G33-D39_), which replaces a longer and more divergent loop region between TMH1-2 in the nonfused AT3 proteins. Further conserved residues are seen in the periplasmic loop between TMH3 and -4, including OafA_S112_, OafA_N113_, and OafA_Y122_. Together, these observations suggest shared key residues in both AT3-only and AT3-SGNH fused proteins and possible adaption of AT3 domains in AT3-SGNH fused acetyltransferases toward their function together with the fused SGNH domain.

### Site-directed mutagenesis combined with *in situ* functional analysis of OafA identifies functional residues within the AT3 domain.

**FIG 3:**
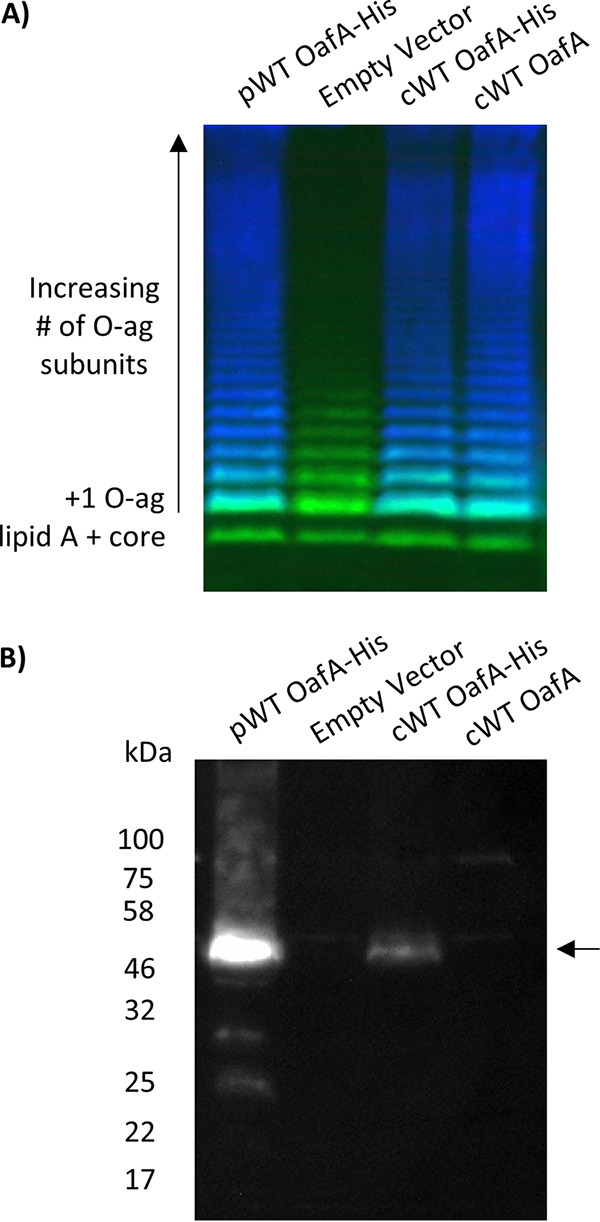
O-antigen acetylation and OafA expression from plasmid and chromosomally expressed protein. (A) LPS immunoblot with crude LPS extracts from Salmonella enterica serovar Typhimurium; LT2 basal O-antigen strain expressing OafA from pBADcLIC plasmid (pWT OafA-His), LT2 wild-type (WT) O-antigen strain with a C-terminal Deca-His tag added to the chromosomal copy of OafA (cWT OafA-His), the same strain with unmodified OafA (cWT OafA), and the LT2 basal O-antigen strain with an empty pBADcLIC plasmid (empty vector). O:5 antibody binding (blue) shows abequose acetylation, and *Salmonella* LPS core antibody binding (green) acts as a loading control. (B) Corresponding anti-His Western blot of insoluble protein fraction for detection of His-tagged OafA. The arrow indicates the full-length OafA protein.

To determine the functional importance of conserved residues in *S.* Typhimurium OafA (OafA-STM), we developed an *in situ* functional assay using a double antibody LPS immunoblot. The assay quantifies both the level of acetylated abequose (O:5) and the amount of LPS based on the O-antigen core (Fig. 3). His-tagged OafA, or mutated versions thereof, were expressed in *trans* in a strain that lacks all O-antigen modification genes, including *oafA* (strain 293) (see Materials and Methods; Table S2). Levels of abequose acetylation in these strains were determined with LPS immunoblotting from the signal obtained with serotype antibody, and protein expression was also confirmed (Fig. 3, Fig. S2). We validated this approach by comparing abequose acetylation to both chromosomal His-tagged OafA and wild-type OafA using the in *trans* system. This showed that despite a higher level of protein in the in *trans* system (Fig. 3B), a comparable level of abequose acetylation was obtained in all strains (Fig. 3A, Fig. S2).

10.1128/mBio.01364-20.2 FIG S2 Functional analysis of OafA membrane-bound domain point mutants *in situ*. (Left) LPS western blot with crude LPS extracts from Salmonella enterica serovar Typhimurium basal O-antigen strain expressing OafA point mutant variants in (A) the membrane domain and (B) the periplasmic domain. O:5 antibody binding (blue) shows abequose acetylation, and *Salmonella* LPS core antibody binding (green) acts as a loading control. (Right) Corresponding anti-His Western blot for expression of His-tagged OafA. The arrow indicates the full-length OafA protein. Download FIG S2, PDF file, 0.7 MB. Copyright © 2020 Pearson et al. 2020 Pearson et al. This content is distributed under the terms of the Creative Commons Attribution 4.0 International license.

10.1128/mBio.01364-20.9 TABLE S2 Molecular biology materials. Bacterial strains and primers used in this study. Primers for cloning of OafA and OafB constructs and creation of OafA point mutant variants on the pBADcLIC_WT-OafA plasmid. Amp, ampicillin 100 μg/ml; Kan, kanamycin 50 μg/ml. Download Table S2, PDF file, 0.6 MB. Copyright © 2020 Pearson et al. 2020 Pearson et al. This content is distributed under the terms of the Creative Commons Attribution 4.0 International license.

**FIG 4:**
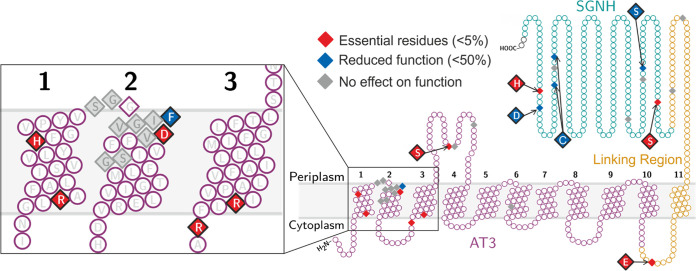
Summary of mutagenesis analysis of *S.* Typhimurium OafA. A diamond shape indicates residues that were mutated, cysteine residues were mutated to serine, and all other residues were mutated to alanine. Results relate to the percentage O-antigen acetylation compared to the wild type; mutants that caused loss of protein expression are diamond shaped but not colored (G34A).

Twenty positions in the membrane-bound domain of OafA were individually engineered to replace the wild-type amino acid with alanine. The level of O-antigen acetylation *in situ* as a result of mutant protein expression is summarized in Table 1 and Fig. 4, and the data are shown in Fig. S2. Point mutants that gave <1% O-antigen acetylation signal in relation to the wild type were considered to be inactive, and those with <50% O-antigen acetylation signal were considered to have significantly reduced activity. For all mutant proteins except G34A, there was detectable full-length protein on the Western blot, sometimes in addition to degradation products (Fig. S2). Assay validation experiments indicate that the levels of full-length mutant protein are in excess of wild-type levels and thus should be sufficient to confer detectable abequose *O*-acetylation.

**TABLE 1:** Summary of site-directed mutagenesis analysis of the transmembrane domain of OafA^*a*^

Mutant	O:5 signal intensity compared to WT (% ± SEM)	Position	Reason for mutation
R14A	0.07 ± 0.04	TMH1	Specifically conserved in AT3-SGNH proteins
			
H25A	0.33 ± 0.18	TMH1	Conserved in TMH1 across all aligned proteins
S32A	105.25 ± 30.89	Periplasmic loop and TMH2	XGG-F/Y-XGV-D/P/V-X motif found to be conserved in AT3-SGNH fused acyltransferases. In the first periplasmic loop between TMH1-2
G33A	119.17 ± 18.72	Periplasmic loop and TMH2	
G34A	1.36 ± 0.88^*b*^	Periplasmic loop and TMH2	
F35A	19.24 ± 2.70	Periplasmic loop and TMH2	
I36A	101.47 ± 22.72	Periplasmic loop and TMH2	
G37A	118.13 ± 22.11	Periplasmic loop and TMH2	
V38A	86.38 ± 12.73	Periplasmic loop and TMH2	
D39A	0.31 ± 0.07	Periplasmic loop and TMH2	
V40A	121.28 ± 23.82	Periplasmic loop and TMH2	
S45A	98.18 ± 24.30	TMH2	Conserved in SG in TMH2
G46A	99.59 ± 22.01	TMH2	
			
R69A	0.10 ± 0.04	TMH3	RXXR motif previously identified as critical for function
R72A	0.07 ± 0.02	TMH3	
			
S112A	0.24 ± 0.09	TMH3-4 Periplasmic loop	Conserved in periplasmic loop between TMH3-4 in AT3-SGNH fused proteins
N113A	93.79 ± 14.92	TMH3-4 Periplasmic loop	
Y122A	85.76 ± 7.58	TMH3-4 Periplasmic loop	
G202A	74.14 ± 10.70	TMH6	Conserved transmembrane glycine
			
E325A (linker)	4.84 ± 1.13	TMH10-11 cytoplasmic loop	Conserved after TMH10 in all AT3-SGNH fused proteins

a Dark gray shading, point mutants with <1% O:5 signal intensity; Light gray shading, point mutants with <50% O:5 signal intensity.

b No OafA protein expression detected. Values represent the average of 2 biological repeats with 3 technical replicates.

The arginine and histidine residues in the R/K-X_10_-H motif (OafA_R14_ and OafA_H25_) are essential for function. These residues are predicted to be on the same surface of the alpha helix with spacing similar to the predicted distance between the 3′ phosphate and the thioester bond of one coenzyme A molecule (∼19 Å). Thus, we hypothesize that these residues provide a potential acetyl-CoA interaction site within the AT3 domain. The 100% conserved glycines (OafA_G46_ and OafA_G202_) could be replaced with alanine with no detriment. As expected, both arginines in the TMH3 RXXR motif (OafA_R69,R72_) (9, 57) were essential for OafA function (Table 1, Fig. 4).

We next examined the unique aspects of the AT3 domains among the AT3-SGNH fused proteins. Of the conserved GG-F/Y-XGV-D/P/V motif and flanking residues, mutation of OafA_F35_ and OafA_D39_ caused significant reduction and complete loss of OafA activity, respectively. OafA_S112A_ mutation also caused complete loss of OafA activity (Table 1, Fig. 4). AT3-only acetyltransferases do not contain an 11th TMH, but a glutamate residue after the C-terminal end of TMH10 (OafA_E325_) is invariant across AT3-SGNH protein sequences; mutation of this residue (OafA_E325A_) resulted in significant reduction in OafA activity (Table 1, Fig. 4). Thus, AT3-SGNH-specific conserved residues in the AT3 domain are inherently involved in the mechanism of action of OafA.

### OafB-SPA^long^ has an extended SGNH-like fold.

To gain an understanding of the mechanism of OafA and OafB, both domains must be analyzed; thus, *in vitro* analysis of the SGNH domain was conducted. Structural analysis of the SGNH domains and periplasmic linking regions of OafA and OafB were used to gain insight into the functional adaptations of an SGNH domain fused directly to an AT3 domain. We expressed and purified residues 366 to 609 from OafA (OafA-STM^C-long^) and residues 377 to 640 from *S.* Paratyphi OafB (OafB-SPA^C-long^), which have 31% sequence identity (Fig. 1A). Although OafB-SPA has not been experimentally characterized in the literature, *S.* Paratyphi O-antigen rhamnose can be acetylated (58), and OafB-SPA has 78% sequence identity to the experimentally characterized OafB-STM rhamnose acetyltransferase (9).

No diffracting crystals of OafA-STM^C-long^ were obtained; however, crystals diffracting to a 1.1-Å resolution were obtained for OafB-SPA^C-long^, with a single molecule in the asymmetric unit. The structure could not be solved by molecular replacement using a number of known SGNH structures but was solved using Fragon (59) with a 14-residue ideal polyalanine α-helix as the search model and refined to an R_work_/R_free_ of 13.6/14.9% (Table S3).

10.1128/mBio.01364-20.10 TABLE S3 X-ray crystallography data and statistics for the structure of OafB-SPA^C-long^. Values in parentheses correspond to the highest resolution shell unless otherwise stated. Download Table S3, PDF file, 0.4 MB. Copyright © 2020 Pearson et al. 2020 Pearson et al. This content is distributed under the terms of the Creative Commons Attribution 4.0 International license.

**FIG 5:**
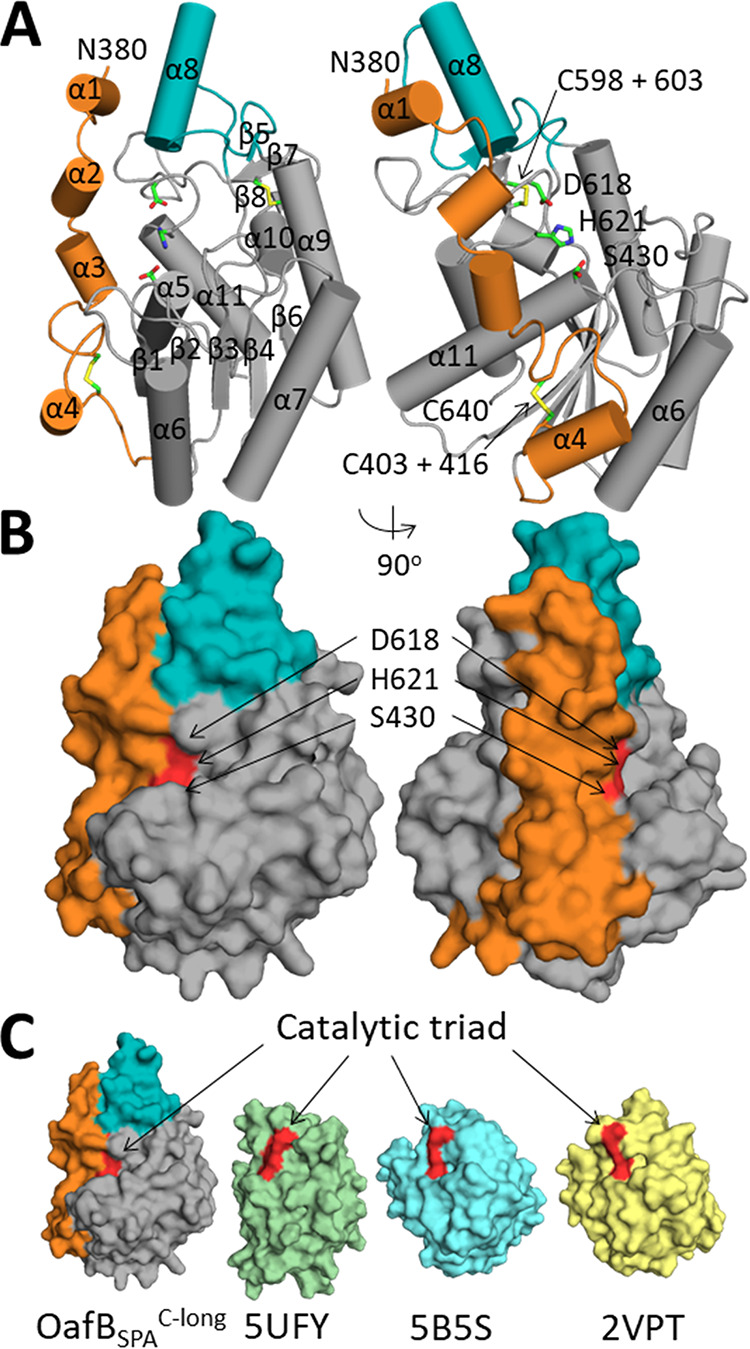
Analysis of the crystal structure of OafB-SPA^C-long^. (A) Cartoon representation of OafB-SPA^C-long^ with helices and sheets numbered, with the additional helix (α8) colored teal and SGNH-extension colored orange. Catalytic residues and disulfide bonds are shown as sticks and are labeled. (B) Surface representation of OafB-SPA^C-long^ with coloring as above and the catalytic triad colored red. (C) Surface representation of OafB-SPA^long^, PDB 5UFY, PDB 5B5S, and PDB 2VPT.

The core structure of OafB-SPA^C-long^ resembles an SGNH domain with an α/β/α hydrolase fold consisting of five central β-strands surrounded by six α-helices (Fig. 5A). Two disulfide bonds are seen in the structure (Fig. 5A) and were verified using mass spectrometry. The closest structural homologues to OafB-SPA^C-long^, as identified by the DALI server, are carbohydrate esterases from Talaromyces cellulolyticus (Protein Data Bank [PDB] 5B5S) and Clostridium thermocellum (PDB 2VPT); each has a root mean square deviation (RMSD) of 2.5 Å over 207 and 201 backbone residues, respectively.

**FIG 6:**
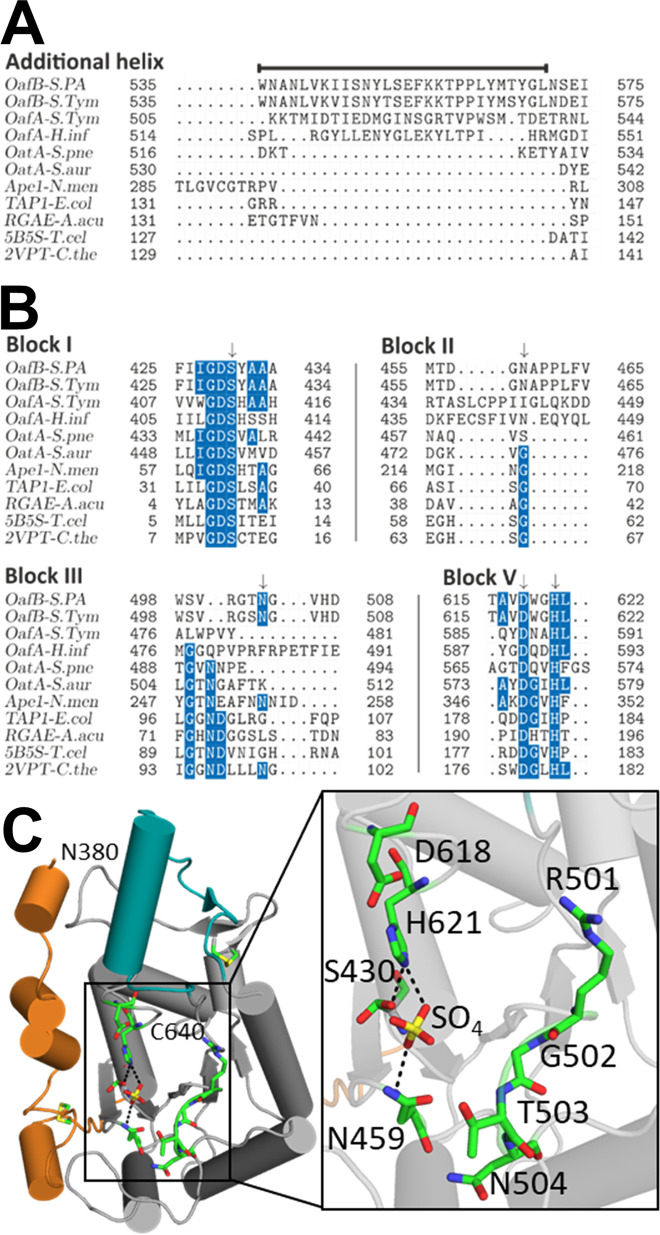
Analysis of additional helix and catalytic triad residues (A and B) Structure-based sequence alignments of additional helix (A), indicated by a line above the sequence, and blocks I to V (B) with residues conserved in >50% of sequences highlighted blue; catalytic and oxyanion hole residues are indicated by an arrow. Abbreviations and details of sequences used are in Materials and Methods. (C) Catalytic triad and potential oxyanion hole residues are shown as sticks; hydrogen bonds to cocrystallized sulfate ion are shown as dotted black lines.

The first clear difference between OafB-SPA^C-long^ compared to its closest structural homologues and the only other SGNH domain from a fused acyltransferase with a solved crystal structure, OatA-SGNH (PDB 5UFY) (60), is that the structure is significantly larger, at ∼36,000 Å^3^, than OatA-SGNH at ∼23,000 Å^3^, which is more similar to the size of the two most closely related structures of the carbohydrate esterases (PDB 2VPT is ∼26,000 Å3 and PDB 5B5S is 27,000 Å3). This additional volume in the fold is contributed by two separate noncontiguous parts of the structure, the first being helix α8, which comprises 10% of the SGNH domain volume (Fig. 5). A structure-based alignment of related SGNH domains indicated that the sequence forming this additional helix is only present in AT3-SGNH domains involved in acetylation of LPS O-antigens (Fig. 6A, Fig. S3) and so is missing on OatA. Second, and most significantly, the region that connects the end of TM11 and the start of the sequence of other known SGNH domains (residues 377 to 421) is clearly structured and forms a long extension of the SGNH domain that we now term the SGNH extension (SGNH_ext_). The SGNH_ext_ interacts extensively with the SGNH domain covering 1,500 Å^2^ of the SGNH domain, including interactions with helix α8; 38 amino acids of the SGNH domain interact with 32 (of 48) residues in the extension. Removal of the most N-terminal half of the SGNH_ext_ (OafA-STM^C-short^ and OafB-SPA^C-short^ [Fig. 1A]), results in a decrease in melting temperature of 5.7°C in OafA and 8.9°C in OafB, suggesting that the SGNH_ext_ has a stabilizing effect on the SGNH domain (Fig. S4). These observations show that OafB-SPA^C-long^ forms an extended SGNH-like fold with an additional helix, and the periplasmic portion of the linking region is structured and interacts with the SGNH domain.

10.1128/mBio.01364-20.3 FIG S3 Structure-based sequence alignment of OafB, OafA, and the closest structural homologues. Residues conserved in >50% are highlighted in blue, and catalytic and oxyanion hole residues are indicated by an arrow. Abbreviations and details of the sequences used are in Materials and Methods. Download FIG S3, PDF file, 0.1 MB. Copyright © 2020 Pearson et al. 2020 Pearson et al. This content is distributed under the terms of the Creative Commons Attribution 4.0 International license.

10.1128/mBio.01364-20.4 FIG S4 Melting curves of OafA-STM^C-long^, OafA-STM^C-short^, OafB-SPA^C-long^, and OafB-SPA^C-short^, with melting temperatures of OafA-STM^C-long^ = 63.8°C, OafA-STM^C-short^ = 58.1°C, OafB-SPA^C-long^ = 58.9°C, and OafB-SPA^C-short^ = 50.0°C. Download FIG S4, PDF file, 0.4 MB. Copyright © 2020 Pearson et al. 2020 Pearson et al. This content is distributed under the terms of the Creative Commons Attribution 4.0 International license.

### Catalytic residues of OafB-SPA^long^ resemble a typical SGNH domain with an atypical oxyanion hole.

SGNH domains are usually characterized by the presence of four blocks of sequence containing conserved residues, block I (GDS), block II (G), block III (GxND), and block V (DxxH) (where x is any nonproline residue) (22). The structure-based sequence alignment was used to identify conserved residues in the SGNH domain of fused acyltransferases (Fig. 6B, Fig. S3). The typical SGNH catalytic triad, consisting of serine (block I), aspartic acid, and histidine (block V), is conserved in the sequence of both OafA and OafB. *In situ* functional analysis of catalytic triad mutants OafA_S412A_ and OafA_H590A_ showed almost complete loss of function, whereas OafA_D587A_ showed reduced activity (Table 2, Fig. S2). This is consistent with analyses of typical catalytic triad activity in other SGNH proteins (61, 62).

**TABLE 2:** Summary of site-directed mutagenesis analysis of the periplasmic domain of OafA^*a*^

Mutant	O:5 signal intensity compared to WT (% ± SEM)	Reason for mutation
C383,397S (linker)	107.40 ± 26.80	Conserved disulfide bonding pairs
C439,453S	185.06 ± 54.63	Conserved disulfide bonding pairs
C567,572S	49.98 ± 4.33	Conserved disulfide bonding pairs
S437A	45.59 ± 3.42	Potential oxyanion hole residue
E569A	99.87 ± 7.01	Conserved between most C-term Cys pair
S412A	0.36 ± 0.26	SGNH domain catalytic triad residues
D587A	10.13 ± 1.70	SGNH domain catalytic triad residues
H590A	0.87 ± 0.62	SGNH domain catalytic triad residues

a Dark gray, point mutants with <1% O:5 signal intensity; light gray, point mutants with <50% O:5 signal intensity. Values represent the average of 2 biological repeats with 3 technical replicates.

While the catalytic triad is conserved in both proteins, the oxyanion hole residues, glycine (block II) and asparagine (block III), are not (Fig. 6B). Analysis of the structure-based alignment of the block II region (Fig. 6B, Fig. S3) reveals that the conserved glycine is replaced by an asparagine in OafB (OafB_N459_). The structure of OafB-SPA^C-long^ shows OafB_N459_ to be within hydrogen-bonding distance of a cocrystallized sulfate ion (Fig. 6C), suggesting that OafB_N459_ could interact with bound substrate and participate in oxyanion hole formation. Homology modeling of OafA-STM^C-long^ based on the structure of OafB-SPA^C-long^ (Fig. S5) suggests that the OafA_S437_ side chain or OafA_L438_ is most likely to replace the block II glycine in the oxyanion hole. This was supported by the *in situ* abequose acetylation assay, which shows that OafA_S437A_ has significantly reduced activity in comparison to wild-type OafA (Table 2, Fig. S2), consistent with the decrease in activity seen on mutation of the oxyanion hole residues in other SGNH domains (60, 61, 63).

10.1128/mBio.01364-20.5 FIG S5 Comparison of potential oxyanion hole residues in OafA and OafB. (A) Homology model of OafA-STM^C-long^ (yellow) modelled on the structure of OafB (gray, extension in orange and additional helix in teal). Catalytic triad and potential oxyanion hole residues are shown as sticks. Residues indicated with OafA first. Both the Ser 437 side chain and the Leu 438 backbone amide are in close proximity to catalytic triad and active site sulfate. (B) Sequence alignment of OafA from Salmonella enterica serovar Typhimurium and OafB from the Typhimurium and Paratyphi A serovars of Salmonella enterica from OafA residues 410 to 450. Alignments were carried out using T-coffee with default settings. The red box highlights predicted replacements for catalytic block II glycine. Download FIG S5, PDF file, 0.5 MB. Copyright © 2020 Pearson et al. 2020 Pearson et al. This content is distributed under the terms of the Creative Commons Attribution 4.0 International license.

The GxND motif (block III), where Asn is typically involved in oxyanion hole formation (20), is not evident in OafA or OafB in the structure-based alignment (Fig. 6B). OafB-SPA^C-long^ contains a GTNG motif (OafB_G502-G505_) close to sequence block III (Fig. 6B), but the side chains of these residues are oriented away from the catalytic triad (Fig. 6C). These observations suggest that, although OafA and OafB display the typical catalytic triad of an SGNH domain, their oxyanion hole arrangement is atypical.

### The SGNH_ext_ confers acceptor specificity.

The structured region that extends the OafB SGNH domain (SGNH_ext_) appears to occlude the active site and results in significantly lower solvent-accessible surface area (SASA) of the catalytic triad residues (40 Å) than in OatA, 2VPT, and 5B5S (132 Å, 110 Å, and 126 Å, respectively) (Fig. 5C). Removing the 22 most N-terminal residues from the structure of OafB-SPA^C-long^ (OafB-SPA^C-short^; Fig. 1A) increases the SASA of the catalytic triad residues of OafB to 107.9 Å.

To assess the potential consequences of an occluded active site for substrate specificity, assays were carried out for OafA and OafB containing the full SGNH_ext_ (OafA-STM^C-long^ and OafB-SPA^C-long^) and those with half the SGNH_ext_ (OafA-STM^C-short^ and OafB-SPA^C-short^) (Fig. 1A). *In vitro* catalytic activity was first confirmed for all constructs via their ability to hydrolyze the ester substrate p-nitrophenyl acetate (pNP-Ac) (Fig. S6), an assay commonly used to test SGNH domain function (64, 65). This activity suggests that all four proteins are correctly folded and catalytically active regardless of the presence or absence of the SGNH_ext_ residues covering the active site (Fig. S6).

10.1128/mBio.01364-20.6 FIG S6 *In vitro* acetyl-esterase activity of C-terminal OafA and OafB assessed by hydrolysis of pNitrophenyl acetate (pNPA). Solid line, active protein; dashed line, heat-treated protein. Error bars = SEM, *n* = 3. Some error bars are obscured by point markers. “C-long” constructs comprise the SGNH domain with full SGNHext, and “C-short” constructs comprise the SGNH domain with fewer SGNH_ext_ residues to expose the SGNH domain active site. See Fig. 1 for details of the C-terminal OafA and OafB constructs. Download FIG S6, PDF file, 0.5 MB. Copyright © 2020 Pearson et al. 2020 Pearson et al. This content is distributed under the terms of the Creative Commons Attribution 4.0 International license.

**FIG 7:**
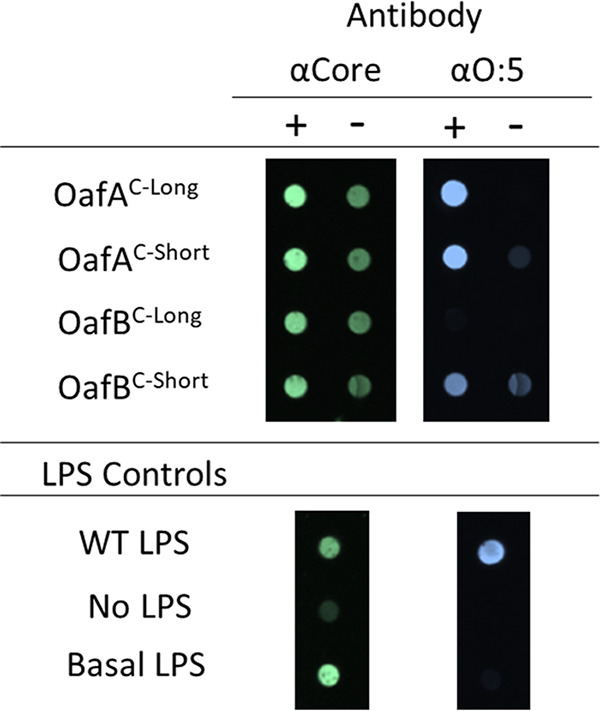
Effect of SGNH_ext_ length on substrate specificity of C-terminal OafA and OafB. Dot blot for acetylated abequose (αO:5, blue) on basal Salmonella enterica serovar Typhimurium LPS after incubation with purified C-terminal OafA and OafB and pNPA as an acetyl group donor; 10 μM OafA and 20 μM OafB were used in these reactions. αCore antibody (green) was used as a loading control. WT acetylated LPS was used as a positive control. +, Active protein; –, heat-treated protein. Representative of *n* = 3 repeats. “C-long” constructs comprise the SGNH domain with full SGNH_ext,_ “C-short” constructs comprise the SGNH domain with fewer SGNH_ext_ residues to expose the SGNH domain active site. See Fig. 1 for details of the C-terminal OafA and OafB constructs.

To assess whether SGNH_ext_ affects the *in vitro* acceptor substrate specificity of OafA-STM^C-term^ and OafB-SPA^C-term^ proteins, purified proteins were incubated with pNP-Ac (acetyl group donor) and unmodified *S.* Typhimurium LPS (Path993; Table S2) as the acceptor substrate, and O:5 antibodies were used to probe for O-antigen abequose acetylation. Abequose is the native acceptor sugar for OafA, whereas OafB acetylates rhamnose *in situ*. A positive signal for O:5 antibody binding is gained after incubation with OafA-STM^C-long^ and OafA-STM^C-short^ (Fig. 7). Thus, OafA-STM^C-long^ and OafA-STM^C-short^ are able to acetylate their native substrate in solution. In contrast, acetylation of the nonnative acceptor substrate by OafB occurs only in the absence of the OafB SGNH_ext_ (OafB-SPA^C-short^) (Fig. 7). First, these results support our working model that the SGNH domain performs the last step in the transferase reaction, the transfer of the acetyl moiety to the acceptor carbohydrate. Furthermore, these results strongly indicate that the acceptor substrate specificity of this SGNH domain is constrained by the cognate, structured SGNH_ext_.

### Evolutionary support for an interaction between the AT3 domain and the SGNH domain.

**FIG 8:**
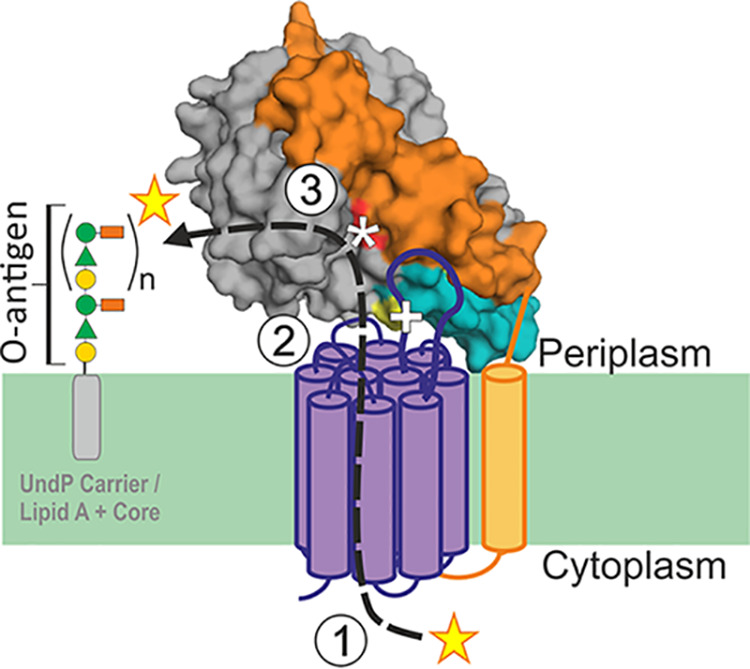
Refined model of AT3-SGNH fused O-antigen acetyltransferases. Periplasmic SGNH_ext_ (orange) is structured, therefore positioning the SGNH domain (gray) close to the AT3 domain (purple); this orients the additional helix (teal) in close proximity to the AT3 domain with interactions between the two domains as proposed by the coevolution analysis. These observations result in the current hypothesis: (1) Cytoplasmic acetyl group donor interacts with conserved Arg in TMH1, the acetyl group is processed and transferred to the periplasmic side of the inner membrane, and this process involves catalytic His residue of TMH1. (2) Conserved Asp and Ser mediate transfer of acetate to the SGNH domain. (3) SGNH domain catalyzes addition of the acetate to specific O-antigen monosaccharide. The active site of the SGNH domain is highlighted by an asterisk, and interaction site is highlighted by a plus sign (+).

The discovery that the “linker” region that is present between the more clearly defined AT3 and SGNH domains is, in fact, a long structured component of the SGNH domain means that the SGNH is much more constrained and proximal to the membrane than initially proposed if this region was a long flexible linker. The discovery that there are residues in the AT3 loop between TMH3 and -4 that are only conserved in the AT3-SGNH fused proteins suggests potential protein-protein contacts between the two domains during catalysis. To test this hypothesis, we used a coevolution analysis of the OafA-B type acetyltransferases to assess whether there was any evidence for correlated changes in the two domains consistent with a physiological interaction (Fig. S7a). While there are many correlated changes within the two separate domains, a significant correlated change was observed between residues 95 and 97, located in the periplasmic loop between TMH3 and -4 of the AT3 domain (Fig. 8) and residues 542 and 545 to 546, which form a surface-accessible patch (Fig. S7b) on the additional helix (α8) of the SGNH domain (Fig. 8). This predicted interaction further informs our refined topological model of these AT3-SGNH acetyltransferases (Fig. 8).

10.1128/mBio.01364-20.7 FIG S7 (A) Predicted contact map for OafB based on a correlated mutation analysis using the RaptorX Web server. The horizontal/vertical line marks residue 377, which forms the boundary at the end of the AT3 domain. High-confidence interactions occur within the AT3 domain (top left) and the SGNH domain (bottom right), while a single high-scoring interaction between the AT3 (93 to 97) and SGNH (524 to 546) is marked (bottom left). (B) Structure of OafB-SPA^C-long^ with residues (542 to 546) predicted to interact with the acyltransferase domain (blue). The extension is colored orange, the additional helix colored teal, and the catalytic triad is colored red. Download FIG S7, PDF file, 0.1 MB. Copyright © 2020 Pearson et al. 2020 Pearson et al. This content is distributed under the terms of the Creative Commons Attribution 4.0 International license.

## DISCUSSION

AT3 domain-containing proteins (PF01757) are a ubiquitous family of proteins involved in diverse carbohydrate modifications across the domains of life. Prokaryotic members of this family play roles in modification of antibiotics and antitumor drugs, as well as initiation of microbial symbioses with plants (15, 66, 67) (Table S1). In bacterial pathogens, such as Salmonella enterica, Listeria monocytogenes, Haemophilus influenzae, and Streptococcus pneumoniae, these proteins are implicated in acetylation of extracytoplasmic polysaccharides, which can have significance for interactions with phage and hosts and can affect virulence and antibiotic resistance (24, 32, 36, 38). The current experimentally characterized AT3 domain-containing carbohydrate *O*-acetyltransferases display SGNH-fused or AT3-only domain architecture. Although both AT3 and SGNH domains display broad substrate ranges in diverse biological systems, the mechanism of action of both SGNH-fused and AT3-only acetyltransferases is largely unknown.

Previous understanding of AT3-SGNH fused acetyltransferases was obtained by *in situ* functional assays and structure-function assessment of the SGNH domain (9, 17, 60). Here, expanded bioinformatic analysis with a set of 30 experimentally characterized bacterial AT3 acetyltransferases, including AT3-only and AT3-SGNH fused protein sequences, which perform a range of biological functions (Table S1), revealed commonalities and key differences. For example, an R/K-X_10_-H motif in TMH1 is shared across all the bacterial AT3 acetyltransferases studied (Fig. 2) and is also highly conserved across all AT3 domain proteins in the Pfam database (29), strongly suggesting that these are critical catalytic residues relevant to the whole protein family.

OafA_R14_ and OafA_H25_ within this motif were essential for activity (Table 1, Fig. 4) and are predicted to be at opposite ends, but on the same surface, of the TMH1 helix (arginine toward the cytoplasmic side), providing a potential interaction site for the proposed acetyl group donor acetyl-CoA. Although cytoplasmic acetyl-CoA has not been confirmed as the donor for O-antigen acetylation, it occupies a central role in bacterial metabolism and is a prominent source of acetate in bacterial cells (68, 69). Arginine residues were implicated in binding of the 3′ phosphate of acetyl-CoA in other acetyltransferase proteins (70), and conserved histidine residues in the soluble mitochondrial carnitine O-acyltransferase coordinate the thioester bond of acyl-CoA with the carnitine acceptor to catalyze the acyl-transfer reaction (71). Significantly, the equivalent residue was discovered as a natural histidine to tyrosine point mutation that decreased function of the *Streptococcus pneumoniae* capsule acetylation protein WcjE in clinical isolates (72).

A similar role for a conserved intermembrane histidine residue has also been suggested for membrane-bound O-acyltransferases containing an MBOAT (InterPro IPR004299) rather than AT3 domain (73). These observations support a role of the R/K-X_10_H motif in coordinating a cytoplasm-derived acetyl-CoA molecule within the membrane-bound AT3 domain for transfer of the acetyl group to the SGNH domain, consistent with our model (Fig. 1). AT3 domain-containing proteins are implicated in transferring a wide range of acyl groups such as succinate, isovalerate, and propionate (67, 74, 75); these can all be carried by coenzyme-A. The proposed mechanism of acetyl donor interaction would provide a potential conserved mechanism for transfer of any of these acyl substituents, supporting the idea that the TMH1 arginine and histidine are fundamentally important for the mechanism of all AT3 domain-containing acyltransferases.

Residues specifically conserved in the AT3 domains of AT3-SGNH fused proteins (OafA_F35_ and OafA_D39_ in TMH2 and OafA_S112_ between TMH3 and -4) are located toward the periplasmic side of the AT3 domain (Fig. 2); we suggest these are likely to be important for interaction with the O-antigen substrate or SGNH domain for acetyl group transfer. In contrast to the essential nature of OafA_S112_ in the periplasmic loop between TMH3 and -4, no functional residues have been identified in the equivalent region of S. flexneri Oac (an AT3-only O-antigen acetyltransferase) (57). Conversely, the invariant glycine residue OafA_G46_, which was critical in S. flexneri Oac (Oac_G53_) (Fig. S1) (31, 57), could be replaced by alanine without affecting the function of OafA. These observations suggest a divergence between AT3-only and AT3-SGNH fused proteins. The location of critical residues specific to the AT3-SGNH fused proteins further suggest that this divergence occurs at the point of acetyl group transfer to the acceptor substrate.

This study demonstrates that the SGNH domain of OafA is able to acetylate the abequose of the O-antigen of *Salmonella in vitro* without the presence of its cognate fused AT3 domain. This supports the predicted role for SGNH in the final step of acetyl group transfer to the acceptor substrate in fused acetyltransferases (Fig. 1B). In agreement with this, in the two-component PatA/PatB peptidoglycan acetyltransferase system, PatB, a soluble SGNH protein, is responsible for transfer of the acetyl group onto the peptidoglycan substrate (62). Moynihan and Clarke hypothesized that PatA (an MBOAT protein, not an AT3) is responsible for transporting the acetyl group across the membrane, where it is transferred to the acceptor by the soluble PatB protein (62). The membrane-bound PatA MBOAT protein in this system is interchangeable with WecH, an AT3-only acetyltransferase protein (52, 76), giving an example of direct transfer of acetate between a membrane-bound AT3 domain and soluble SGNH domain protein. This supports the mechanistic model of the AT3 domain delivering the acetyl group to the SGNH domain for transfer onto the acceptor substrate in AT3-SGNH fused proteins (Fig. 1B).

Our data demonstrated, for the first time in a fused system, the necessity for the fused SGNH domain in glycan carbohydrate acetylation. However, this poses the conundrum that other closely related systems, such as OacA from *Shigella*, which *O*-acetylates rhamnose in the O-antigen (57), lack either a fused or genetically linked partner SGNH domain. Consequently, either the AT3 domain functions differently or there is a currently undiscovered partner protein.

This study elucidates the structure of the SGNH_ext_ in OafB-SPA^C-long^ and shows that removal of this region results in promiscuity of carbohydrate modification in *in vitro* acetyltransferase reactions (Fig. 7). These findings suggest that the SGNH_ext_ plays a role in determining the specificity of the O-antigen residue to be acetylated. Closer examination of the structure reveals that two tyrosines, Tyr289 and Tyr394, in the SGNH_ext_ sit close to the active site and could potentially be involved in a mechanism to limit off-target acetylation. Inadvertent acetylation of complex carbohydrates could potentially have diverse and undesired biological effects due to the variation of cellular processes that can be affected by acetylation (9, 33, 39, 77–79). Whether this also suggests that AT3 proteins all need a partner domain or protein for substrate-specific transferase activity remains to be determined.

Coevolution analysis predicts interaction between periplasmic loops of the AT3 domain and the SGNH domain of OafB. This is similar to the arrangement of domains seen in PglB, an oligosaccharide transferase from Campylobacter lari (80), with 13 TMH and a periplasmic domain. In PglB the periplasmic domain interacts via periplasmic loops in the transmembrane domain, and both domains are hypothesized to interact with the peptide substrate (80). In our model, the coevolution analysis positions the periplasmic loops of the AT3 domain close to the α8 helix in the SGNH domain, allowing for an interaction with each other and with the acceptor substrate (Fig. 8).

AT3 domain-containing proteins are involved in the modification of a wide range of polysaccharides and influence many host-pathogen interactions. These structural and functional insights can be applied to the well-studied and biotechnologically relevant AT3 proteins, including Nod factor modifications important for plant microbe symbiosis, and antitumor and antibiotic modifying proteins. Furthermore, this work can inform future studies of eukaryotic systems where AT3 domain-containing proteins are involved in regulation of the life span of Caenorhabditis elegans (81) and in *Drosophila* development (82).

## MATERIALS AND METHODS

### Bacterial strains, plasmids, and culture conditions.

Escherichia coli and *S.* Typhimurium strains and plasmids are listed in Table S2. Strains were cultured in Lennox broth (LB; Fisher Scientific) at 37°C with appropriate antibiotic selection unless otherwise stated.

### *In silico* analysis of bacterial AT3 domains to identify conserved residues.

A survey of the literature identified 30 experimentally characterized bacterial carbohydrate acetyltransferases; these sequences were aligned with OafB from *S.* Paratyphi A, using T-coffee (83). Protein accession numbers are in Fig. S1. T-coffee was also used to align the OafA-STM, OafB-STM, and OafB-SPA protein sequences for direct comparison.

Structure-based sequence alignments using PROMALS3D with default settings were carried out with the two closest structural homologues identified using the DALI server and a selection of typical SGNH domains for which structural information is available, OafB-SPA, 1IVN, 4K40, 1DEX, 5UFY, 5B5S, and 2VPT. Five additional representative sequences of OafA, OafB, and OatA were included (A0A0H2WM30, STMMW_03911, Q8ZNJ3, NTHI0512, and Q2FV54).

### Coevolution analysis.

A multiple sequence alignment of AT3 SGNH domain fused proteins was constructed using the MUSCLE alignment tool based on 1,188 full-length sequences from the UniProt reference proteomes. This alignment was used to construct a profile hidden Markov model (HMM) to detect further homologues in the UniProt reference proteome set as well as within the MGnify protein sequence set. We required that all matches to this profile-HMM had a sequence and domain threshold of 27 bits. We also required that the sequence matched >90% of the HMM match states to ensure that homologues with only one of the two domains were not included in the alignment.

A total of 2,713 homologues were identified from the UniProt reference proteome set, and 9,757 homologues were identified from the MGnify metagenomics sequences. A large sequence alignment was constructed using OafB as the master with no indels with all the sequence matches aligned to it using the hmmalign package and a custom Perl script to format the alignment for contact prediction. The alignment was submitted to the RaptorX contact prediction server (84).

### Molecular biology.

Primers (Sigma-Aldrich) are listed in Table S2. Mutations were introduced into the OafA sequence (pMV433 as the template), which had been cloned into pBADcLIC using blunt-end ligation, placing the gene under the control of an arabinose-inducible promoter. Mutants were confirmed by sequencing. Plasmids were electroporated into *S.* Typhimurium strain 293 (Table S2) for analysis of activity.

All *oafA*-STM and *oafB*-SPA sequences for protein expression were cloned into pETFPP_2 (Technology Facility, University of York) using in-fusion cloning (Clontech) to add a 3C-protease cleavable N-terminal His-MBP tag. Plasmid pMV433 (Table S2) was used as the template for creation of expression plasmids encoding the protein sequence for OafA-STM^C-long^ (residues 366 to 609) and OafA-STM^C-short^ (residues 379 to 609). *oafB*-SPA (UniProt A0A0H2WM30), amino acid residues 377 to 640 for OafB-SPA^C-long^, was codon-optimized for E. coli and synthesized by Genewiz in a pUC57-Kan vector. This vector was then used as a template for the sequence encoding OafB-SPA^C-short^ (residues 399 to 640); see Table S2 for the primers used.

### *In situ* functional analysis of OafA variants.

All *in situ* functional analyses of OafA variants cloned into pBADcLIC were carried out in strain Path293 (23) (Table S2). Strains for the *in situ* functional analysis were cultured at pH 7.0 in 100 mM sodium phosphate-buffered LB at 37°C in a baffled conical flask with shaking at 200 rpm. Overnight cultures were diluted 100-fold and grown for 16 h. Samples were normalized to an optical density at 600 nm (OD_600_) of 3.0 per ml for LPS and protein extraction.

### Crude LPS sample preparation.

The method was adapted from Davies et al. (23). First, 1 ml of OD-normalized (OD_600_, 3.0) overnight culture was pelleted for 5 min at 16,000 × *g*. Cell pellets were resuspended in 100 μl LPS sample buffer (60 mM Tris-HCl, 1 mM EDTA, pH 6.8) containing 2% (wt/vol) SDS and then boiled at 100°C for 5 min. Then, 400 μl of LPS buffer was used to dilute the solution before RNase (Roche) and DNase (Sigma) treatment at 37°C for 16 h. Samples were then treated with 100 μg proteinase K for 16 h at 50°C, and 7.5 μl of crude LPS extracts were run on 1.0 mm Tricine SDS poly acrylamide gel electrophoresis (TSDS-PAGE gel) for analysis by immunoblotting.

### Detection of OafA protein expression for *in situ* assays.

First, 1 ml of OD-normalized culture was pelleted for 5 min at 16,000 × *g*. Soluble and insoluble fractions were isolated from cell pellets using BugBuster solution (Novagen) following the manufacturer’s instructions for soluble protein extraction. The insoluble pellet was resuspended in 75 μl of sample buffer (10% [vol/vol] glycerol, 1% [wt/vol] SDS, 10 mM Tris-HCl [pH 7.2], 0.06% [wt/vol] bromophenol blue, 3% [vol/vol] β-mercaptoethanol), heated to 60°C for 10 min, and centrifuged for 10 min at 16,000 × *g*. Then, 10 μl of insoluble fraction samples was loaded onto a 12% acrylamide 1.0-mm SDS-PAGE gel for analysis.

### Immunoblotting.

First, 7.5 μl of crude LPS extracts was run on 1.0 mm Tricine SDS polyacrylamide gel electrophoresis (TSDS-PAGE) gel for analysis by immunoblotting. The TSDS-PAGE-separated LPS samples and SDS-PAGE-separated protein samples were transferred onto Immobilon-P polyvinylidene difluoride (PVDF) membrane (Merck-Millipore). For His-tagged protein detection, the primary antibody was Tetra·His antibody (1:1,000) (Qiagen; in 3% [wt/vol] bovine serum albumin [BSA] Tris-buffered saline [TBS]), and the secondary antibody was goat anti-mouse IgG-HRP (1:10,000) (Sigma-Aldrich; in 5% [wt/vol] milk TBS). The blot was developed using Luminata Classico Western HRP substrate (Merck-Millipore). For LPS detection, O:5 serotyping antibody (1:10,000) (Statens Serum Institute; 40272) and *Salmonella* core antigen (1:200) (Insight Biotechnology; 5D12A) were used as the primary antibodies and goat anti-rabbit IgG StarBright Blue700 (1:5,000) (Bio-Rad) and goat anti-mouse IgG (H+L) DyLight 800 (1:5,000) as the respective secondary antibodies. LPS antibodies were diluted in 5% milk phosphate-buffered saline with Tween 20 (PBS-T). The ChemiDoc MP imaging system (Bio-Rad) and Image Lab (Bio-Rad) were used for image capture and analysis. The *in situ* activity of OafA mutant relative to the wild-type protein was derived from quantification of the O:5 signal in each lane, standardized to the intensity of the single O-antigen repeat band for the *Salmonella* core signal on LPS immunoblots. Assay validation demonstrated that <1% of the O:5 signal with respect to the wild type was within the background variation. Variation increased significantly for signal intensities in the higher range; therefore, the O:5 signal recorded between 50 and 100% relative to wild type was not interpreted further.

### Expression and purification of OafA-STM^C-term^ and OafB-SPA^C-term^.

The pETFPP_2 vectors containing the inserted OafA-STM^C-term^ and OafB-SPA^C-term^ constructs (Fig. 1) were transformed into Origami (Novagen) E. coli for protein expression. Protein expression was carried out as described by Gruszka et al. (85) without the addition of protease inhibitor. The proteins were purified using immobilized metal affinity chromatography with a HisTrap FF column (GE Healthcare) utilizing a His-tag, followed by size exclusion chromatography after His-tag removal, as described by Wojdyla et al. (86); purified protein was eluted in 20 mM Tris-HCl (pH 7.5) and 100 mM NaCl.

### Melting temperature of OafA and OafB SGNH domains.

The melting temperature of SGNH domains was determined using NanoDSF with a protein concentration of 1 mg/ml in 20 mM TrisHCl (pH 7.5) and 100 mM NaCl. Proteins were heated from 20°C to 95°C with a heating rate of 2°C/min. The fluorescence at 330 and 350 nm was measured every 0.05°C.

### *In vitro* acetylesterase activity assay.

The catalytic activity of OafA and OafB C-terminal constructs was confirmed by acetyl esterase activity using pNP-Ac as a substrate. One hundred μl of enzyme solution (10 μM OafA-STM^C-term^, 40 μM OafB-STM^C-term^, or 0.04 U/ml acetyl xylan esterase) or appropriate control buffers were added to relevant wells of a 96-well plate and incubated at 37°C for 10 min prior to addition of pNP-Ac. Then, 100 μl of 1 mM pNPA in the corresponding buffer was then added to matching sample and control wells and immediately placed into a plate reader incubated at 37°C. Absorbance at 405 nm was measured at *T* = 0, and then at 5 min intervals.

### *In vitro* abequose acetyltransferase activity assay.

Crude LPS extracted from OafA-negative *S.* Typhimurium strain LT2 (Path993) was heated at 100°C for 20 min to inactivate the proteinase K (see above). Heat-treated LPS was mixed 1:1 with KPi buffer (200 mM NaCl, 50 mM potassium phosphate buffer [pH 7.8]). Next, 10 μM OafA-STM^C-term^ and 20 μM OafB-SPA^C-term^ were incubated at 4°C in the LPS-KPi mixture with 4 mM pNP-Ac dissolved in ethanol (4% [vol/vol] final concentration in reaction). Samples of the reaction mix were taken after specified time points and inactivated by boiling for 10 min.

Then, 5 μl of LPS reaction samples were loaded onto methanol-activated PVDF membrane using a Bio-Rad Bio-Dot microfiltration apparatus. The protocol for LPS detection with O:5 serotyping antibodies and *Salmonella* core antigen was followed as per immunoblotting, following removal of the membrane from the apparatus after sample loading.

### Protein structure analysis.

To crystallize OafB-SPA^C-long^, a hanging-drop vapor diffusion method was used with 20 mg/ml OafB-SPA^C-long^ in a drop ratio of 1:1 protein:reservoir solution. After incubation for 24 h at 20°C, crystals grown in 100 mM BisTris (pH 5.5), 0.25 M lithium sulfate, and 25% PEG 3350 were cryoprotected by addition of glycerol to a final concentration of 20% and vitrified in liquid nitrogen.

X-ray diffraction data for crystals of OafB-SPA^C-long^ were collected on beamline I04-1 (Diamond Light Source, UK) at a wavelength of 0.9282 Å using a Pilatus 6M-F detector. Data were integrated with XDS (87) and scaled and merged with AIMLESS (88) via the Xia2 pipeline (89). Fragon molecular replacement (59) used Phaser (90) to place an ideal poly alanine helix of 14 amino acids in length followed by density modification with ACORN (91). ARP-wARP (92) was used for automated chain tracing, and the model was refined using REFMAC 5 (93–98). Manual manipulation of the model between refinement cycles was performed using Coot (99, 100). The final model was evaluated using MolProbity (101) and PDB validate; the secondary structure shown in Fig. 5A was annotated using STRIDE (102).

A homology model of OafA-STM^C-long^ was produced using SwissModel with the structure of OafB-SPA^C-long^ as a template (103–107).

### Data availability.

The atomic coordinates and structure factors have been deposited in the Protein Data Bank (PDB ID code 6SE1).
