# Supplementary material for: Acetylation of Surface Carbohydrates in Bacterial Pathogens Requires Coordinated Action of a Two-Domain Membrane-Bound Acyltransferase
Source: mBio. 2020 Aug 25;11(4):e01364-20. doi: 10.1128/mBio.01364-20 (PMC7448272; doi:10.1128/mBio.01364-20)
Supplement: TABLE S1 [file mBio.01364-20-st001.pdf]

Table S1. Experimentally characterised bacterial AT3 acetyltransferases

Protein Name	SGNH fused	Species	Accession number	Reference
OafA STM	Y	*Salmonella*	WP_000639473	(Slauch *et al.* 1996)
OafB STM (F2GtrC)	Y	*Salmonella*	SIU02679	(Kintz *et al.* 2015)
HI_OafA	Y	*Haemophilus*	AAX87447	(Fox *et al.* 2005)
OatA (LMO129)	Y	Listeria	CAC99369	(Aubry *et al.* 2011)
Lot3 (PurB)	Y	*Neiseria*	WP_002245844	(Kahler *et al.* 2006)
OatA (lp_0856)	Y	Lactobacillus	WP_011101182	(Bernard *et al.* 2011)
OatB (lp_0925)	Y	Lactobacillus	YP_004888877	(Bernard *et al.* 2011)
OatA	Y	*Staphylococcus*	WP_000379821	(Bera *et al.* 2005)
yvhB (OatA)	Y	Lactococcus	WP_021723064	(Veiga *et al.* 2007)
Adr (OatA)	Y	*Streptococcus*	WP_001220853	(Crisóstomo *et al.* 2006)
PglI	Y	Neisseria	WP_003687310.1	(Aas *et al.* 2007; Anonsen *et al.* 2017)
OatB (BAS5308)	N	*Bacillus*	YP_031545	(Laaberki *et al.* 2011)
ExoZ	N	*Rhizobium*	WP_010975904	(Buendia *et al.* 1991)
GumF	N	*Xanthomonas*	WP_011037591	(Katzen *et al.* 1998)
GumG	N	*Xanthomonas*	AAA86375	(Katzen *et al.* 1998)
Lag1	N	*Legionella*	AAA75102	(Zou *et al.* 1999)
NodX	N	*Rhizobium*	P08888	(Davis, Evans and Johnston 1988; Firmin *et al.* 1993)
NolL	N	*Rhizobium*	Q52778	(Pacios Bras *et al.* 2000)
OacA	N	*Shigella*	P23214	(Verma *et al.* 1991)
OacA Bt	N	*Burkholderia*	WP_009895914	(Brett *et al.* 2011)
OacA Bp	N	*Burkholderia*	WP_004545264	(Brett *et al.* 2011)
OacB	N	*Shigella*	NP_706267	(Wang *et al.* 2014)
OacC	N	*Shigella*	EFW62204	(Knirel *et al.* 2014)
OacD	N	*Shigella*	WP_000282635	(Sun *et al.* 2014)
WbaK	N	*Salmonella*	Q54131	(Hong *et al.* 2013)
WbiA	N	*Burkholderia*	WP_004194788	(Brett, Burtnick and Woods 2003)
WciG	N	*Streptococcus*	WP_001230914	(Geno, Saad and Nahm 2017)
WcjE	N	*Streptococcus*	WP_000170108	(Calix and Nahm 2010)
WecH (YiaH)	N	*Escherichia*	P37669	(Kajimura *et al.* 2006)
CmmA	N	Streptomyces	Q70J69	(Menendez et al., 2004)

This list was finalised on 27/02/19
