# Supplementary material for: Acetylation of Surface Carbohydrates in Bacterial Pathogens Requires Coordinated Action of a Two-Domain Membrane-Bound Acyltransferase
Source: mBio. 2020 Aug 25;11(4):e01364-20. doi: 10.1128/mBio.01364-20 (PMC7448272; doi:10.1128/mBio.01364-20)
Supplement: FIG S1 [file mBio.01364-20-sf001.pdf]

Fig. S1. Alignment of characterised AT3 acetyltransferases. Protein sequences are in the same order as Table S1 after *Salmonella* ser. Paratyphi A OafB WP_00400612. SGNH fused acetyltransferases are indicated by a grey box. Asterisk mark residues selected for mutation from this alignment.

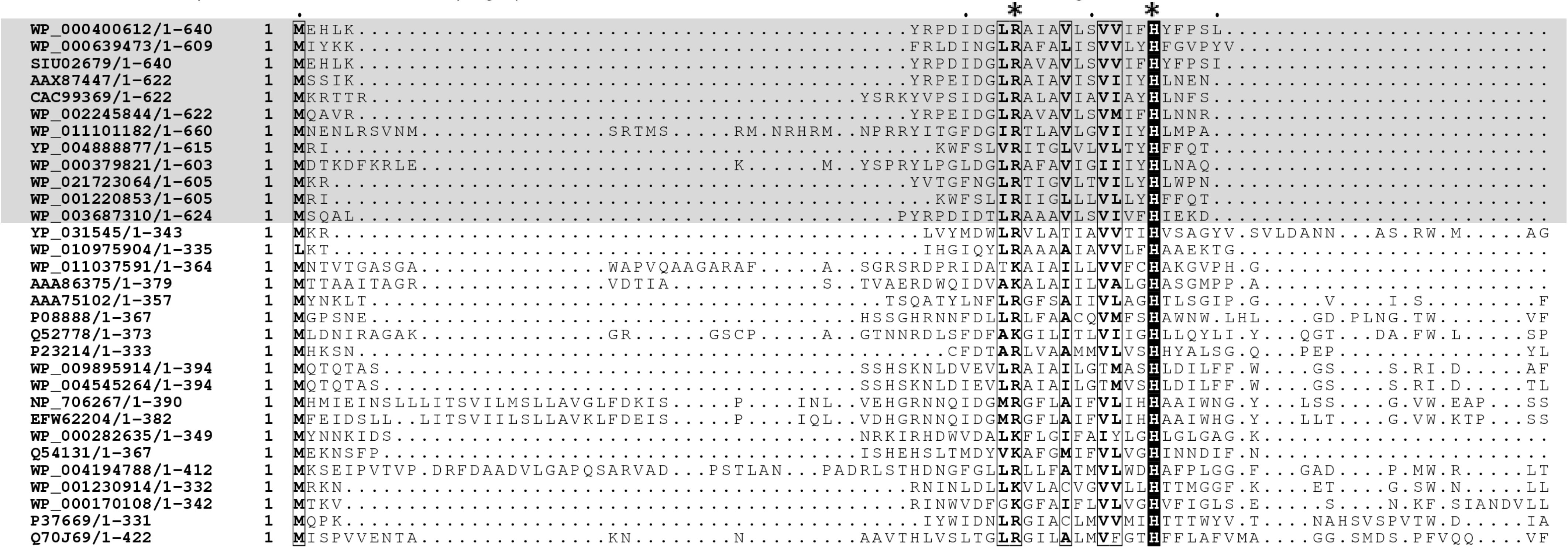

```
                                    *******    **  .           .              .*  *    .           .            .
WP_000400612/1-640   31  ....LPGGFVGVDIFFVISGYLITSIILKSAS.NK.S....FSYLDFYKRRVLRIF.PALSI.VLVSCL.IVGWIY.LFQ.D.DY.KLLG..KH...VF.
WP_000639473/1-609   32  .....SGGFIGVDVFFVISGFLMTGIVLERVD.H......KGVLDFYIARFLRIV.PALVF.AILLLM.IFGLFT.LST.N.EY.EALS..KN...AI.
SIU02679/1-640       31  ....LPGGFVGVDIFFVISGYLITSIILKSAS.SN.S....FSYVEFYKRRILRIF.PALSI.VLISCI.IIGWVY.FFQ.D.DY.KSLG..KH...VF.
AAX87447/1-622       30  ...WLSGGFLGVDIFFVISGFLITGIIITEIQ.QN.S....FSLKQFYTRRIKRIY.PAFIT.VMALVS.FIASVI.FIY.N.DF.NKLR..KT...IE.
CAC99369/1-622       35  ...WAKGGFIGVDIFFVLSGYLITNILLTQWEKNQ.S....LQLKQFWIRRFRRLI.PAVYV.MIVVVV..I.YSV.FFH.P.EILKNLR..GD...AI.
WP_002245844/1-622   30  ...WLPGGFLGVDIFFVISGFLITGIILSEIQ.NG.S....FSFRDFYTRRIKRIY.PAFIA.AVSLAS.VIASQI.FLY.E.DF.NQMR..KT...VE.
WP_011101182/1-660   51  ...SLQGGYLGVPIFFVVSGYLITDILLQDILSRG.H....VRIWRFLGHRMRRLY.PAFVT.MLLGTT..A.YIT.LFQ.R.SLLTNIR..AT...VL.
YP_004888877/1-615   26  ...QFTGGFIGVDIFFTFSGFLITSLMVDEFARSD.N....FKLMAFYGRRFYRIV.PPLFI.AVLLVL.P..LTY.LID.H.DFVTDIG..KQ...VA.
WP_000379821/1-603   41  ...WLSGGFLGVDTFFVISGYLITSLLISEYYRTQ.K....IDLLEFWKRRLKRLI.PAVLF.LICVVL..T.FTL.IFK.P.ELIIQMK..RD...AI.
WP_021723064/1-605   28  ...HVQGGFLGVVLFFVLSGYLVTDSLLREYEKNK.K....INIWQFWGRRLKRLY.PLLIA.IFLLVT..P.YII.IFQ.P.NLWAGLR..SN...FL.
WP_001220853/1-605   26  ...IFPGGFFGVDVFFTFSGFLITALLIEEFSKNN.E....IDLIGFFRRRFYRIV.PPVVL.MVLVTM.P..FTF.LVR.Q.DYVAGIG..GQ...IA.
WP_003687310/1-624   31  ...WLPGGFLGVDIFFVISGFLMTAILLREMS.GG.R....FFLKTFYIRRIKRIL.PAFFA.VLAATL.AGGFFL.FTK.D.DF.FLLW..KS...AL.
YP_031545/1-343      43  N.LFESISRASVPIFVMISGALLLKGTKD............ISVGEFLQKRASKVIIPFIAW.SAIFYA.YGAYAG.YFP.A.S................
WP_010975904/1-335   29  ..HHFTIGAAGVDVFFVISGFIMWVIS.....DRR.S....VTPVEFIADRARRIV.PVYWL.ATGVMV.AG.ALA.GLF.P.NLVLTL...EH...VL.
WP_011037591/1-364   55  ..MTLFAYSFHVPLFFLVSGWLAAGYASRT...........TSLLQTITKQARGLLLPYVVF.YLLGYV.....YW.LLT.R.NI.GE..........K.
AAA86375/1-379       48  ..YKLFAYSFHVPLFFVLSGWVGERFGRRA...........FG.RKTVGKLARTLLIPYVSF.FLVAYG.....YW.ILS.A.VL.NG..........T.
AAA75102/1-357       40  G.KQLPFQSLAVNAFFWLSGFLITYHCITK....K.P....YTFAEYMIDRFCRIY.VIYIP.VLILSV.....FL.LAK.A.DL.ASMPELKE...WV.
P08888/1-367         48  DLL...FSAPGVAIFFLISGFLVTDSYIRS...........SSAASFFVKRSLRIF.PALFV.NIAVME.LA.LLV.TGG.L.NV.T......G...IL.
Q52778/1-373         59  ..YFKSIYMFHMPLFMAISGYLSSGAILR............KSFTQGVGERAMQLLLPMLFW.CTLIWT.LKS.AV.IFP.MKS...............
P23214/1-333         35  F.GFESAGGIAVIIFFSISGYLISKSAIRS...........DSFIDFMAKRARRIF.PALVP.CSILTYFLFGWILNDFS.A.E...............
WP_009895914/1-394   47  ..QRTFFFWGGVDLFFCISGYVITGNLLRTLP.SGRQCGFGAFAIPFWIRRAWRIL.PSAWL.WLAIPL.IL.SVV.ANR.T.GYLGTPT..GNMIDSL.
WP_004545264/1-394   47  ..QRAFFFWGGVDLFFCISGYVITGNLLRTLP.SGRQGGFGAFAIPFWIRRAWRIL.PSAWL.WLAAPL.IL.SVV.ANR.T.GYLGTPK..GNIIDAL.
NP_706267/1-390      77  N.LLANLGQVGVSFFFMITGYLFFSKIIS.....G.D....QDWTRLYVSRLLRLT.PMFIV.SLCLIF.I..IVG..FK.S.GWRM......Q...VS.
EFW62204/1-382       75  N.LLTNLGQVGVSFFFMITGYLFFSKIRS.....S.D....QDWVRLYISRFLRLT.PMFIV.SLCLVL.L..VIG..FK.S.RWSV......H...VS.
WP_000282635/1-349   40  ..LYPFVFSYHVPLFFFAAGFFTIKKN.........D....LSVFDYIKSKFYRLMIPYFTF.AFSILI.I..NTI.NSG.E.TI.DYIY..NH...IY.
Q54131/1-367         39  ...VYYAYLFHMPLFFFIGGVLYKDTRC...............ITNFTAHVIKKQL.PYLIV.TYLIIG.S..IAL.LIN.V.RY.GIHT..GD...AF.
WP_004194788/1-412   79  L.NQDSMGGICVSGFFAISGFLIAKSGMRA............DALQFAWRRCVRIF.PAYWA.VLIVTALCVGPIIHYVQ.A.GTLHGYW..NA...ALG
WP_001230914/1-332   40  A.YLYYLGTYSIPLFFMVNGYLLLGKRE...............ITYL..YILQ.KV.KWILI.TVSSWS.F..IVW.LFK.R.DF.TTN..........
WP_000170108/1-342   44  F.LIAQIYIFHIPVFFALSGYFFRPVSD...............LKEFWYYAKK.KT.IILGI.PYIFYS.I..IHF.CLQ.K.LA.GAS...V.......
P37669/1-331         44  N.VLNSASRVSVPLFFMISGYLFFGERSA..................QPRHFLRIG.LCLIFYSAIALL...YIA.LFTSI.NM.ELAL..KN...LL.
Q70J69/1-422         59  HVLFSRAGGTAVSCFFLLSGFVLAYIARPG...........DSTRSFYRRRIAKIY.PVHLI.STAVAF.I...LV.SVR.Y.EV.PGM...EV...TL.
```

```
                                   .            **          .             *                  .          .           .                  .
WP_000400612/1-640    108   SGSFFI.........SNF.....TLWS.E.....S....G.Y.......FD...SKSY.L.KPLLHLWSLGIEEQFYIIW......PVVILLCF.RSK.N
WP_000639473/1-609    106   SSLLFY.........SNN.....YYAI.H.....S....S.Y.......FD...SSSE.F.NFLLHTWSLSVEWQFYILY......PLLVIIVK.KLR.F
SIU02679/1-640        108   SGAFFI.........SNL.....TLWS.E.....S....G.Y.......FD...SQSY.L.KPLLHLWSLGIEEQFYILW......PIVILLCF.KSK.Y
AAX87447/1-622        108   LAIAFL.........SNF.....YLGL.T.....Q....G.Y.......FD...LSAN.E.NPVLHIWSLAVEEQYYLIF......PLILILAY.KKF.R
CAC99369/1-622        113   ASFFYV.........SNW.....WFIFHN.....V....S.Y.......FD...SFGL.P.SPLKNLWSLAIEEQFYLIW......PAFLLVFL.KWV.K
WP_002245844/1-622    108   LSAVFL.........SNI.....YLGF.Q.....Q....G.Y.......FD...LSAD.E.NPVLHIWSLAVEEQYYLLY......PLLLIFCC.KKT.K
WP_011101182/1-660    129   TNLVYV.........YNW.....FEINHG.....Q....S.Y.......FD...RFNG.E.SPFTHLWSLSIEGQYYLFW......PLVIGILM.VIF.K
YP_004888877/1-615    104   AALGFT.........TNY...F..EIA.T.....G....G.S.......YE...NKFI.P.HLFVHTWSLAVEMHFYILW......GLIAWLVA.KIS.R
WP_000379821/1-603    119   AAIFYV.........SNW.....WYISQN.....V....D.Y.......FN...QFA..I.EPLKHLWSLAIEEQFYLLF......PLVITFLL.HRF.K
WP_021723064/1-605    106   SAIFSV.........QNW.....WQISQG.....S....S.Y.......FA...DIAG.A.SPFKHIYYLSIEGQFFILW......PLLLIVLL.KFV.K
WP_001220853/1-605    104   GVLGFM.........TNF...Y..ELL.T.....G....G.S.......YE...SQFI.P.HLFVHNWSLAVEVHYYILW......GLAVWFLS.KQA.K
WP_003687310/1-624    109   TALGFA.........SNL.....YFAR.G.....K....D.Y.......FD...PAQE.E.KPLLHIWSLSVEEQFYFVF......PILLLLVA.RKS.L
YP_031545/1-343       109   ................L.....KQGI.......K....H.F..............LT.D.TIGGHLWFLYMIVGIYLIT......PLLKVFVK.NAK.K
WP_010975904/1-335    103   ASLFFV.........PAR...............................SP...SSGE.IWPVLVQGWTLNFEMLFYAVF......AGSLFMP.......
WP_011037591/1-364    121   AARWGS.........HPW.....WEPI.......V....S.M.......FT...GVGPDL.YVQPPLWFLPVMLVTVIGY...........VLLR.RWM..
AAA86375/1-379        113   SQSWAG.........HPW.....WHPF.......V....GLL.......WA....NGSSL.YVLPALWFLPALFVATVVY...........LALR.EDL..
AAA75102/1-357        115   ANIFMI.........QHT.....PFNR.I.....F....E.F.......LP...TIPP.L.AQISPLWSIAVEWWLYTLF......GIAFFFHK.SSFAN
P08888/1-367          116   QYLFYF.TVYILTAARIWAVYFTYEPYTM.....S.....GF.......YG.....A.S.DPSGVLWTLTVELTFYLTLP......MLLEIWR.RWK.R
Q52778/1-373          124   ................L.....TDTL.......L....D.L..............S.T.EVIGTYWFIWAAFISFILI......RVLTTF.......N
P23214/1-333          103   ...YFS.........HDI.....VR.........KTISS.IFMSQAPDADITSHLIH.A.GINGSLWTLPLEFLCYIIT......GVAVAHL....K.N
WP_009895914/1-394    134   SAVAQV.........ANF.....HFWS.C.....Y....A.F.......PA...K..T.C.GIDQVYWSLSLEEQCYILL......PVLLYSAS.RRT..
WP_004545264/1-394    134   SAVAQV.........ANF.....HFWA.C.....Y....A.F.......PA...T..T.C.GIDQVYWSLSLEEQCYIVL......PVLLYFAN.RRT..
NP_706267/1-390       147   TEELFVSI.......MKW...L..PFT.ALGMPNI....N.D.......VK...DSFT.I..NAAVTWTLVYEWFFYFSL......PVISALIK.RKV.S
EFW62204/1-382        145   PASLTVSL.......MRW...A..PFT.ALGMPNI....N.G.......VK...DSFT.I..NAAVTWTLVYEWFFYFSL......PVIAALFK.RRV.S
WP_000282635/1-349    111   DIIYGV.........RN.................................N.QFVGTIWFINCLFVIIAID......AI....FR.EIV.K
Q54131/1-367          106   STGLY..........ETV.....KLAI.K.....S....N.F..............HN.N.KMFLTGWFLFAYIFVSILS......VIIIKSIK.RVV.V
WP_004194788/1-412    157   GPLGYI.........TNN.....WRLTIG.....QYGIND.LLRDTTPYGH...SISE.S.VFNGSIWTLIYEAKCYVMV......GLFAMFGL.LTA.H
WP_001230914/1-332    101   ................PI.....KKIV.......G....S.L..............IQ.R.GYFFQFWFFGALILIYLCL......PILRQFLNSKRS..
WP_000170108/1-342    108   ................RV.....PTTI.......H....N.L..............L......NIYRYPLGVSWYLYTLWSILIVYGLLSIVFK.NRK..
P37669/1-331          111   Q...................................................K.PVFYHLWFFFAIAVIYLVS......PLIQV....KN..V
Q70J69/1-422          131   SHVFLV.........QSW...VP....SQ.....W.....YY..............L.TMTGIDWSLSCEAFFYLCFP......VLLPVLS.RAR.N
```

```
WP_000400612/1-640   163  HNR....NIV......LSCAT.IFII...SYA.I....SIF.TM........AS.....D.G....GA...................NYYSP.....A...
WP_000639473/1-609   161  .P.....VGL.......SLSV.ILAM...SLA.I....TLM.RV........TG.....T.K....ED...................IFYLI.....P...
SIU02679/1-640       163  SKR....NIL......LSCAA.IFIV...SYT.I....SVF.TM........AY.....E.G....GA...................NYYSP.....A...
AAX87447/1-622       163  .EI....KVL......FIITL.ILFF..ILLA.T....SFI.PA........NFYKEVLH.Q....PN...................IYYLS.....N...
CAC99369/1-622       169  NPK....LLL......KIVIG.LGLLSAVWMT.I....LYV.PG.........T.....D.P....SR...................VYYGT.....D...
WP_002245844/1-622   163  .SL....RVL......RNISI.ILFL..ILTA.T....SFL.PS........GFYTDILN.Q....PN...................TYYLS.....T...
WP_011101182/1-660   185  KRS....RVF......WFMMI.AAGISAITMA.M....LY..DP.........A.....N.T....NR...................VYYGT.....D...
YP_004888877/1-615   159  RLASGRRALTRFRWGLGLLTTAFAIESFVMMY.F....GAV.GL.........K.....D.F....SP...................VYFSS.....L...
WP_000379821/1-603   174  PRN....IIQ.......TLFI.VSLISLGLMI.V....IHF.IT.........G.....D.N....SR...................VYFGT.....D...
WP_021723064/1-605   162  KRG....RIF......VIANL.LALISAIWMA.I....LFV.PG.........A.....D.P....TR...................VYYGT.....D...
WP_001220853/1-605   159  SNG....QL...KGMVFLLSAVAFLISFFSMF.I....GSF.LV.........T.....S.Y....SS...................VYFSS.....L...
WP_003687310/1-624   164  .RV....QFG.......FLAA.LCAL...SLA.A....SFM.PS........AL...............D...................KYYLP.....H...
YP_031545/1-343      151  .RE....IEY.......FLIL.WLYA...SVV.VNLVKYYY.PI........NF.....NI.........................ELFYVT.....N...
WP_010975904/1-335   146  RNW....RLP......VVSG..LFLALV..IA......GRV.VA.........F.....D.D....AV...................MLTYT.....R...
WP_011037591/1-364   171  .......PPL.......VIAA.VAVV...LAW.F..WMNWF.PL........QH             MR...................LFWGL.....D...
AAA86375/1-379       163  .......SAA.......VLAV.CSLL...VVW.A..WTRWF.PG........LR             LR...................LPFAL.....D...
AAA75102/1-357       171  .RL....IMS.......IL.I.IPAL...LVA.G....YFT.LK........EY...........................................
P08888/1-367         182  .AG....ALV......VAVAA...LG.SWVMA......QHF.NI.........T.....D.K....YNPFLSVT.................A.....G...
Q52778/1-373         160  .RL....SIW.......IISA.S..........AIAVAFA.PI........TL.....SIT....PL..................LKY...........
P23214/1-333         162  .GK....AFI........VIL.LV.....FVS.L....SLIGS.........VS.....E.N....RD...................VMFSIPLWLYP...
WP_009895914/1-394   186  .IM....PIL......CVVV..........LI.Q....VFL.PR                      PI...................LSFLW.....F...
WP_004545264/1-394   186  .IA....PIL......CVVV..........LI.Q....VFL.PR                      PI...................LSFLW.....F...
NP_706267/1-390      208  IY.....M........VMISA.ISLFVFIL..........................................F.FS.....K...
EFW62204/1-382       206  IH.....M........IIISV.LVLVIFLC..........................................YPSK.....K...
WP_000282635/1-349   147  .NN....IVI......LIISL.LSFM..LSQT.V...LNHN.PL.........L.......D....PQ...................WFWNID........
Q54131/1-367         156  .S.....NAL.......LLSV.LVAI...SVL.L....ITV.SI........TY.....L.S....PQ...................YILVK.....DY.K
WP_004194788/1-412   224  .RR....VLL.......AVTV.VAWF...VLA.I....QTINPAFSAQLLPWAG.....D.R....HL...................VQY...........
WP_001230914/1-332   144  .......YLY.......SLSL.LMTI...GLI.F....ELS.NI........LL.....Q.M....PI..................QTYVIQ.....TFRL
WP_000170108/1-342   152  .......SLF.......LVSV.FAYI..FTLF.I....QI..D.......................................IFIVQ.....RT..
P37669/1-331         140  GGK....MLL......VLMAV.IGII...AN..P....NTV.PQ........KI.....D.GFEWLPI...................NLYIN.....G...
Q70J69/1-422         179  .GM....LYA......VSACS.VFLV.FFLPYVVG...HTF.AI.........R.....S......PDPVEMVPTAGYGGPIGYWFAYVFP.....P...
```

```
                                               .                      *  .                     .                                                   .                                                             .
WP_000400612/1-640    199   .SR...FWEL...MAGAIISTL.RFI.GI.NT..SLSKL....M.SL.............LGI.IL.IA..................L...SIT.M..
WP_000639473/1-609    194   .TR...AWEM...LAGGLVYIA.SVR.YK.MP..EWIKH....C.EV.............YGI.VL.IV..................V...AVV....
SIU02679/1-640        199   .SR...FWEL...MAGAIIATL.RFM.GI.KT..SVSKS....M.SL.............IGV.II.IT..................L...SIA.L..
AAX87447/1-622        204   .LR...FPEL...LVGSLLAIY.HNL.SA.SK..QASK..Q..ASNV......IAI.LS...T.LL.LF..................S...CLF.L..
CAC99369/1-622        207   .TR...AFDL...LSGCALAFV.WPF.NRLSP..VVPRKS.KAVLNI......AGT....ISILCF.IL..................F...TAF....
WP_002245844/1-622    204   .LR...FPEL...LAGSLLAVY.GQT.QNGRR..QTAN..G..KRQL......LSS.LC...F.GA.LL..................A...CLF.V..
WP_011101182/1-660    222   .TR...MFAI...LLGSGLAFI.WPS.RELSA..DIANVN.RVTLDI......LGG....ASL.IA.II..................W...IFF.Q..
YP_004888877/1-615    208   .TH...CFPF...FVGGLIGVL.SGI.KA.QG..PIYRWT.TAH.....CNPIIAGAVMLISF.IL.LV..................A...LGD.R..
WP_000379821/1-603    211   .TR...LQTL...LLGCILAFI.WPP.FALKK..DISKKI.VVSLDI......IGI....SGF.AV.LM..................T...LFF.I..
WP_021723064/1-605    200   .TR...FFSL...IMGASLAFV.WPL.NKLSH..KVNKRAVKIAWQL......TIG....LSL.LL.LL..................A...YIF....
WP_001220853/1-605    201   .TH...VYPF...FLGSMLATI.VGV.RQ.TT..SLVKQL.DKIWDLRKTLVVFGGGF...GF.LV.LL..................T.....F.F..
WP_003687310/1-624    195   .LR...ACEM...LVGSLTAVR.MRY.RQQRN.PAVGKR....Y.AA......VGA...LFSA.CI.LS..................A...CLF.A..
YP_031545/1-343       188   .Y....VGYF...LLGYYLSNF.....DI.SK..KWRNI....S.YI..............GG.F.VGF..................I...STF.FI.
WP_010975904/1-335    180   .PV...ILEF...VAGMIIGEF.WLK.GRVPP..L....AVGS.ALF......ACS....LGG.FA.LI..................G...VLG....
WP_011037591/1-364    203   .VL...PVSLCFYALGALLIHV.SPYLPT.SLP....GS....A..L.............VTV.........................VVAALVA
AAA86375/1-379        195   .VL...PVALFFIAVGAWLSRF.AERV.R.ALPAVVWVV....A............................................FPVLAFA
AAA75102/1-357        194   ..V...ALVW...FLGSGCAYH.F.C.NIN...RKYNNH....G.IL......MLS..LITGA.AF.LV...................RFH.V..
P08888/1-367          217   .PT...FWIF...SMGVLARLY.W.H.RV.SK..IFEGK...LLWWL.............AT.....HL..................AITWWVA.G..
Q52778/1-373          189   .T....YPFY...CLGFLFAQP.IG..WQ.NG..VIWRY....K.WI..............FV.VLLSI..................A...A......
P23214/1-333          198   .LR...GLAF...FFGATMAMY.EKS.W......NVSNV....KITV.............VSL.L..AM..................Y...AY.....
WP_009895914/1-394    211   .VR...TDAL...ALGALLALL.QHR.EW.YA..KVEPRTL..ARGL......LSA.IALAGM.CL.IL..................A...ALP.G..
WP_004545264/1-394    211   .VR...TDAL...ALGALLALL.QHR.DW.YA..KVEPRPL..GRGL......ISA.IALAGM.CI.VL..................A...ALA.G..
NP_706267/1-390       228   .IH...IASF...LFGL.LAFL.LNK.SKIVN..GIAK....AK.....VT.....PIIITA..IM.V......................F.E..
EFW62204/1-382        227   .IH...VISF...LFGL.LASY.SNN...YVH..RVAK....AK.....IT.....PIMIIA..LL.S......................Y.E..
WP_000282635/1-349    182   .SAMAYWWLL...PLGRCMF.LEL....T.RD..RFFGK......SK......I.......GF.IV.FSITAIMSAYQLLNQKPLLFKII...SI.....
Q54131/1-367          191   LNF...ICQV...LTGMS.FYI.FGY.VI.RN..QIYNL....L.NF.............YVF.IL.L....................TVIL...
WP_004194788/1-412    264   ......GTIF...LIGSSAAAY.SKS.L......PISDK.....LGA.............FAV.VV.YL.....................I.....
WP_001230914/1-332    180   WTW...FFYY...LLGGYIAQF.TIE.EI.ES..RFKNW....M.KI.............VSI.LL.LL..................I...SPI....
WP_000170108/1-342    178   LVW...GICF...FLGSVLSEI.HFD.KI.........NL....K.KF.............LFF.FV.LF.....................DFIYMFA
P37669/1-331          179   .DT...FYYI...LYGMLGRAI.GMM.DT.QH..K...A....L.SW.............VSA.AL.FA..................T...GVF.I..
Q70J69/1-422          234   .MR...MAEF...VIGISLAVL.VRR.GA.WR..........GPGV.............AAC.LV.LC..................AIGWGVN.L..
```

```
WP_000400612/1-640   241  ..ID..EKM..........................SFPGY............IA..IIP...V.LGAS.LI.........IASN.GNDLVVSKLLSVRPV
WP_000639473/1-609   235  ..IL..HSN....G.....................YWPSY............SA..LAP...V.LGAS.MV.........ILAN.KQ.NS..LFTSNRIA
SIU02679/1-640       241  ..IN..EKM..........................AFPGY............IA..IIP...V.IGAS.LI.........IASN.GNDWIASKILSFKPI
AAX87447/1-622       250  ..MN..NDI....A.....................YIPGI............TL..ILP....CIFTA.LI.........IHTT.SQNNIIKLCLSNKVI
CAC99369/1-622       257  ..VSEYQPF..........................LYRGG............LL..FVA...I.LGVI.MI.........ATIS.HPASYLSKIFSFKPL
WP_002245844/1-622   251  ..ID..KHN....P.....................FIPGM............TL..LLP....CLLTA.LL.........IRSM.QYGTLPTRILSASPI
WP_011101182/1-660   272  ..MSGQSDF..........................TYRGG............ML..LFT...V.LSTV.LV.........ATVA.HPASHLNRVLTNPLF
YP_004888877/1-615   262  ..MKFDALQ..........................TYQGG............LL..LAT...V.LAGI.MI.........YGAR.LLHDHTPNLKEPRWM
WP_000379821/1-603   261  ..VGDQDQW..........................IYNGG............FY..IIS...F.ATLF.II.........AIAV.HPSSLFAKFLSMKPL
WP_021723064/1-605   250  ..MPAQGTF..........................TYYGG............MW..LAS...L.ASVI.MV.........ALVA.HPSLPTNKLFSNPVF
WP_001220853/1-605   255  ..VKFTYLF..........................AYLIG............FL..LAS...L.AALA.MI.........LAAR.VLHEKTHHIQESKII
WP_003687310/1-624   243  ..YS..EQT....A.....................YFPGP............AA..LIP....CLAVA.AL.........IYFN.HYEHPLKKFFQWKIT
YP_031545/1-343      226  .TYF..YTV....KA.NG.......QLEQFW..YGYFAPG............VV..LMA.................IGLFIFFKYAFQKS..ERELPLLF
WP_010975904/1-335   225  ..LPFDELT............................TGP............LA..VLL...V.IG...............VLS.LEANGC..VRALSLP
WP_011037591/1-364   244  WL...AGVN....G...........RIDVNM..LEFGRQH............AVFLLSA...V.AGSL.MV.........I....CA.AR..LVQEWTWL
AAA86375/1-379       235  WGGV.AAMN....G...........QVDVNN..LQFGKSS............LLFLIAS...L.LGTA.MT.........L....CI.AY..FMQGWRWL
AAA75102/1-357       238  ..LK..HSL....M.....................NMYDL............QL..VIPG...CIFLY.SL.........LLLL.STT.KLTKK.IELIS
P08888/1-367         260  ..TS.AAFI..........................SI..N............NA...AP...V.DAFR.IA.........VLAG.LVL.SAAHSLPRPNL
Q52778/1-373         226  .FIC..FLG....WG.K.........E.TYA..Y....NNLVLIHDEQSAKQVF..LMFSGSLAASAV.AMQSMFQCWRL.......VYS..TRVARFVA
P23214/1-333         235  ........A..........................SYGKG............ID.YTMT...C.YILV.SF.........STIA.ICT.S...VGD.PLV
WP_009895914/1-394   262  ..RL..ASI....P.....................FHTGL............IA..LVS....CIMVW............IASY.GKSYTMPDGAVKRVL
WP_004545264/1-394   262  ..YL..ARI....P.....................FHTGL............IA..LMS....CVMVW............IASY.GRGYTMRDGAVKRVL
NP_706267/1-390      269  ..MTY.FKT..........................TYAPL............PL..IL......CGIT.FI.........IIAS.GC..DLYGILRLNIT
EFW62204/1-382       266  ..MIY.FPT..........................TYAPL............PL..II......CGVC.FI.........LIAS.GC..DFYGILRLNLT
WP_000282635/1-349   241  ..FN..............A.........DIISS..SYIQAI...........NT.IITT....VGLIIFN..........I....FI.AK..IICGNDFI
Q54131/1-367         231  ...Y..VSK....SYGFSTQTIM...S......WSYYPDG............LI..MSV...I.NALI.GI......YAVFFISLLI.TR..GMKEIKLL
WP_004194788/1-412   297  ........S..........................LFKGG............YL..LLG...Y.PAM...V.........YAIL.WLACRL..PRWARRI
WP_001230914/1-332   222  .............ILFFIAKTIYHNLF......AEYFYDT............LF...VK...V.STLG.IF......LTILML..T..LN..ESR.RESI
WP_000170108/1-342   218  WFLF..YEV....GS.K.........K.DY...VSYINPG............LW..GIA...F.IVCV.LV......AFAIFPK.ME.KN..FPKTFLYF
P37669/1-331         218  ..IS..RGTLYELQW.R.........G.NFADTWYLYCGP............MV..FIC...A.IA.L.LT.........LV....KNTL..DTRTIRGL
Q70J69/1-422         275  ..HL.DSYL..........................PLAAG............ML...IP...F..ALL.IP.........ALST.ADVNSAWSPLRWKPL
```

```
                                   .             .                    .                                           .
WP_000400612/1-640   282   VFFGLISYPLYLWHW....PIYSF.YRSIFAG.........S.P...........DY.H.........EL....T....LL.....LL.LSFFLAIL...
WP_000639473/1-609   274   QWVGKISYSVYLWHW....PVIVA.MKHYDIEF........S.A...........IN.I.........FF....G...........VI.VSFALGDI...
SIU02679/1-640       282   VFIGLISYPLYLWHW....PVYSF.YRSIFSG.........S.P...........ST.N.........EL....L....IL.....MA.LALVLAIL...
AAX87447/1-622       292   VFIGKISYSLYLYHW....IFIAF.AYYITGE.........KQI...........NN.Q.........SI....A....IV.....II.LTIIFSVL...
CAC99369/1-622       300   RWIGTRSYGIYLWHY....PIITL.TTPVLEI.........TQP...........NI.W.........RA....I....LQ.....VA.ATFIIAEL...
WP_002245844/1-622   293   VFVGKISYSLYLYHW....IFIAF.AHYITGD.........KQL...........GL.P.........AV....S....AV.....AA.LTAGFSLL...
WP_011101182/1-660   315   KYVGQRSYGIYLYQF....PVMIF.YETKVKNI........GDH...........LL.L.........NS....L....IE.....VA.LILIVTEL...
YP_004888877/1-615   305   TFLADVSYSVYLYHW....PLYVI.FSHMMV..........N.W..............L.........AA....L....LT.....TV.LSIILSAL...
WP_000379821/1-603   304   LIIGKRSYSLYLWHY....PIIVF.VNSYYVQ.........GQI...........PV.Y.........VY....I....IE.....IL.LTALMAEI...
WP_021723064/1-605   293   EYIGSRSYGIYLWQL....PVFAF.AEAKVL..........APT...........AW.Y.........NL....I....WQ.....LA.LILILTEL...
WP_001220853/1-605   298   SFLADTSYAVYLFHW....PFYII.FSQLTS..........N.L..............L.........AV....L....LT.....LI.CSYGFASL...
WP_003687310/1-624   285   VAAGLISYSLYLWHW....PILAF.MRYIG.PD........N.L...........PP.Y.........SP....A....AA.....IV.LTLAFSLI...
YP_031545/1-343      276   RFINQASLGIYILHF....FLLNN.LLYMVFPK.V......NNH...........VH.A.........IL....A....IP.IN..VT.ITIVLSMV...
WP_010975904/1-335   259   GLLGDASYSIYLWHT....FAISV.VAKAGLAI........GLG...........AP.A.........TM....F....AA.....VL.SGTLIGIA...
WP_011037591/1-364   291   QWIGRNTLLILCTHM....LVFFV.LSGVAALA........GGF...........GG.A.........RP....G....L.GWAIFVTLFALVASVP...
AAA86375/1-379       284   RWIGANTLLILGTHT....LVFLV.VTSVVVRT........GVI...........DR.K.........LI....G....TPVWALALCAFAIAACIP...
AAA75102/1-357       279   AFLAFISYTLYLSHE....PIRRV.VSTFIEPT........N.L...........KR.G.........FL....I....CG........VCIACATI...
P08888/1-367         298   LRRQDLSYGIYLYHM....LVMHT.LIAIGW.V........G.............HW.W.........LW....I....VE.....PV.GTVALAAL...
Q52778/1-373         290   VQLGQSTLLLYLVQG....AVFRL.MDLIQFGE.V......WNL...........TTRI.........TF....A....TV.LG..VA.I.VVIAMA...
P23214/1-333         268   KGRFDYSYGVYIYAF....PVQQV.VINT..L.........HMG...........FY.P.........SM....L....LS.....AV.TVLFLSHL...
WP_009895914/1-394   302   VYFGSRSYAIYLIHV....PVYRL.TREIWERVATTPSAIDGSF...........TL.R.........FA....F....TA.....IP.IVLLLAEL...
WP_004545264/1-394   302   VYFGSRSYAIYLIHV....PVYRL.TREIWERIVITPSTIGGSF...........TL.H.........FA....F....TA.....IP.IVLVLAEL...
NP_706267/1-390      307   RKLGETTYSVYLLHG....IFLYCLMTWIIPN.........N.Y...........TE.N.........TF....IILVSTT.....AF.LITFTSCL...
EFW62204/1-382       304   RKLGETTYSIYLLHG....VFLYCVMTWVIPS.........N.Y...........SN.Y.........FF....IMLISIT.....AF.CVTLLSCI...
WP_000282635/1-349   281   VRAGRNTLNICGMEYITKLFIPMA.LAIIGFSV........TIP...........NP...........IC..........A.....II.YTCICVYVSDK
Q54131/1-367         285   KMIGQNSRAIMAYHL....LVYVI.LDIIAS...........IL...........GD.Y...SLSGTDVYDNHFI....TK.WSVPVY.IALGLLLP...
WP_004194788/1-412   330   GSRNDYSYGIYVFGF....LVQQV.LAYVGAY.........KYG...........FV.F.........YL....A....AS.....VF.FTFICAWF...
WP_001230914/1-332   270   VSLSNQTMGVFIIHT....YIMKV.WEKVLG.F........NFV...........GA.Y.........LL............F...AL.FTLSVSFI...
WP_000170108/1-342   269   TKYGKDSLGIYILHA....PICSM.IRILMLKV........G.I...........NS.V.........FL....H....VV.VG..IV.LGWYLSIL...
P37669/1-331         268   GLISRHSLGIYGFHA....LIIHA.LRTRGIE.........L.K...........NW.P.........IL....D....II.W...IF.CATLAASL...
Q70J69/1-422         315   VWFGEISLSFYVSHL....LVQEE.LFSRLWNL........GMRAGLLPAPLPVLSW.WVAVL.....SF....L....AQ.....FA.LAVLAAWL...
```

```
                            .       *     .                               .           .               .            .
WP_000400612/1-640  329 ....TYYLI....EKPLRNSRS.....................KY.ITAILLTLS.VFG.TGLI.....GAFIFHI.NGVKDR..EINKS.AGEYA....
WP_000639473/1-609  320 ....SYRTI....ENTLRKRVK....................LQF.NIVLFS.....ST.LALC.....LFVMFT..KGVS......FRF.S...D....
SIU02679/1-640      329 ....TYFLL....EKPLRHSRK.....................KS.ITTIILAVV.VFG.SGIF.....GIVTYSM.NGIKER..SVNKS.AGEYA....
AAX87447/1-622      340 ....SYYLI....EQPIRKSKL.....................NF.KQSFLYIYF.IPS.LLLL.....GFNLYK..RQT....................
CAC99369/1-622      348 ....SFRFI....ETPIRKNGFINYFKGFKDKNYFIWKNKPVGKW.L..SIAGVVA.VLAIFTL..GMSNVLSVNT.NAEKQQ.TSVKTTTSTPDEK...
WP_002245844/1-622  341 ....SYYLI....EQPLRKRKM.....................TF.KKAFFCLYL.APS.LILV.....GYNLYA..RGI....................
WP_011101182/1-660  364 ....SYRFI....ENPMRHYDYSRLLVDFKD.....FLRK..PKF.NRVTTAIVAL.TTVLFVI..TAVGFVQQPS.KAEANKKTELQKT.IAANSKAAD
YP_004888877/1-615  349 ....SYYVL....EPVIAGKRA.....................HVFGHTVTWRQL.QLP.VI..TAGVLLAVNTGVVVKNAPQLSTLEQN.L........
WP_000379821/1-603  352 ....SYRFI....ETPIRKKGFKAFAFL........PKKK..GQF.ARTVLVILLLVPSIVVLS..GQFDALGKQH.EAEKKEKKT.EFKTTKKKVV...
WP_021723064/1-605  340 ....SYRLI....ELPTQRFDYSNILGILQN..F.VREKG..WKL.KKNILPMLIS.GLALISL.....GFIIFSP.PSPHDQ.RVIEEK.IMAQQV...
WP_001220853/1-605  342 ....SFYVL....EPWIAGKNT.....................PIV.QTLRPLPY.IHA.ILAAGTGILTIIVCTV.TLLAPQVGAFETD.L........
WP_003687310/1-624  332 ....SYHCI....EKPFKKWKG.....................SF.AQSVLWIYA.LPM.LVLG.....AGSFFA..MR.....................
YP_031545/1-343     328 I...TLVLQ....RIPVVKK................................................................................
WP_010975904/1-335  308 ....AYMML....ERPLLRRGRAR............................................................................
WP_011037591/1-364  345 L...RWFLM.RFA.......................................................................................
AAA86375/1-379      339 M...RAVLVRRALDVGIE..................................................................................
AAA75102/1-357      325 ....IAYLL....ENKHLVVR...............................................................................
P08888/1-367        344 ....SWALI....EQPAMKLRT.....................SL.VA....................................................
Q52778/1-373        342 I...RSIAR....NLGYVSR................................................................................
P23214/1-333        314 ....SWNLV....EKRFLTRS...............................................................................
WP_009895914/1-394  359 ....NYRFV....ETPLRLHGT.....................RV.A.....................................................
WP_004545264/1-394  359 ....NYRFV....EAPLRVYGA.....................RL.A.....................................................
NP_706267/1-390     359 ....TFKLI....ETPFIKLTK.....................QTT.T...L................................................
EFW62204/1-382      356 ....TFKLI....ELPFINITK.....................QTA.M...K................................................
WP_000282635/1-349  334 IGHWLSRTV....GGPFLIK................................................................................
Q54131/1-367        345 L...IFSIL....KQKVIGK................................................................................
WP_004194788/1-412  378 ....SWHLI....EKRALALKD..............................................................................
WP_001230914/1-332  316 I...VGMLM....KIPYFNR................................................................................
WP_000170108/1-342  319 A...TYILK....KIPFLNI................................................................................
P37669/1-331        316 ....LLSML....VQRI...................................................................................
Q70J69/1-422        379 ....LHRLV....EQPLVRRLRP....................KD.VP....................................................
```

```
                                                                     .         .                  .                .
WP_000400612/1-640  384 .......................................SVTDVYNYYKYGELLRGGICHSVQ..L.......TA.AISNG......CIKNGKHNIFIIG
WP_000639473/1-609  364 .......................................TLKQVVEYRMDNSPWRPDICFLNPDQD.......YS.AFSKC......QDKMTEKSFVVWG
SIU02679/1-640      384 .......................................SVTNVYDYYKYGELLRGGICHSVL..L.......KD.AISNG......CIKNSRNNIFIIG
AAX87447/1-622      381 ........................................IRAEKEHI.........................EQ.SIPVS......NENHYPAKVIILG
CAC99369/1-622      429 ..............KDDKKEDKATKD.........KEADSNKAS.EQK..........................ETQKPDNKNKSAATPKTIITQTVAIG
WP_002245844/1-622  382 ........................................LKQEHLRP.........................LP.GAPLA......AENHFPETVLTLG
WP_011101182/1-660  443 KKNAAALKRQKAAQAAAASSKKVATEKMQTKQAEAKLNSKQKQVEKEY..........................D.LKP......QVVLAMANTDLTAIG
YP_004888877/1-615  407 ............................................WVSGIYQDIDKIGTTHEAV..LAAVTPKKKQ.ATPK.......GDQEKAPGVSIIG
WP_000379821/1-603  426 ..............KKDKQEDKQTAN.........SK...................................................EDIKKSSPLLIG
WP_021723064/1-605  414 ..............ALQKKQLA.........EANNKVPMSLKAVAEKY..........................K.VQP......VVAEKASQMNVLALG
WP_001220853/1-605  400 ............................................TVNGLKQAATNIGQTKVMA........ERAD.ANS..........LGIADGTMLIG
WP_003687310/1-624  372 ........................................LPFMAQYDRLGLTRSNTSCHNNT..G.......KQ.CLW........GDTEKQPELLVLG
YP_031545/1-343         ....................................................................................................
WP_010975904/1-335      ....................................................................................................
WP_011037591/1-364      ....................................................................................................
AAA86375/1-379          ....................................................................................................
AAA75102/1-357          ....................................................................................................
P08888/1-367            ....................................................................................................
Q52778/1-373            ....................................................................................................
P23214/1-333            ....................................................................................................
WP_009895914/1-394      ....................................................................................................
WP_004545264/1-394      ....................................................................................................
NP_706267/1-390         ....................................................................................................
EFW62204/1-382          ....................................................................................................
WP_000282635/1-349      ....................................................................................................
Q54131/1-367            ....................................................................................................
WP_004194788/1-412      ....................................................................................................
WP_001230914/1-332      ....................................................................................................
WP_000170108/1-342      ....................................................................................................
P37669/1-331            ....................................................................................................
Q70J69/1-422            ....................................................................................................
```

```
WP_000400612/1-640   429  DSYAAALFNGLSHYIDNKGSDYIIS.QMTD.GNAPPLFVD...GKDDLQRSVITLNNNRINEIKRVQPEVVLLT..WSVRGTNGVH..D.KKLAIDALSL
WP_000639473/1-609   411  DSHAAHLMPGLKSVFGNSL...NIT.QRTA.SLCPPIIGL...QKDDRP.YCKDINDMVAKEISDNKPTTVLMSALWPV...YP.........MRDYLPE
SIU02679/1-640       429  DSYAAALYNGLSSYIKNNNEKYVIS.QMTD.GNAPPLFVS...GKDDLQRDVGSINADRIKEIGIVKPEIVLLT..WSVRGSNGVH..D.KKLAIEALSL
AAX87447/1-622       409  DSHSSHLEAFLNYV.GNKE.GWKAD.IFKDKFECSFIVNE...QYQLDP.NCQSVWQKDSQY.KAIFISAFYDL.RMGG...QPVPRFR.PETFIEP..D
CAC99369/1-622       479  DSVMLDIEPYLKEAVPNITIDGLVGRQLRD...AITTATG....YKK...............FNSENSSVILE..L...GTNGPF..T.E.......DQ
WP_002245844/1-622   410  DSHAGHLRGFLDYV.GSRE.GWKAK.ILSLDSECLVWVDE...KLADNP.LCRKYRDEVEKA.EAVFIAQFYDL.RMGG...QPVPRFE.AQSFLIP..G
WP_011101182/1-660   510  DSVLLDVSSDLQDVIPGTVVQGRVGRQVTE...VPGIINS....LKS...............QGQLAHNVLLN..I...GTNGTI..T.D.......DQ
YP_004888877/1-615   453  DSVTLGTRSYLGDHVANSNIDAEGDRTMNL...AYKVMMN....QQR...............SHTLRQYVVIC..I...GTNALD..DYE.......EQ
WP_000379821/1-603   452  DSVMVDIGNVFTKKIPNAQIDGKVGRQLVD...ATPIVKS...QYKD...............YAKKGQKVVVE..L...GTNGAF..T.K.......DQ
WP_021723064/1-605   458  DSVMVAASTNLQEVFPHMYIDAAVGRQAES...LDTDLTN....AKS...............KMPNPDAYLIG..L...GTNGTI..K.E.......GE
WP_001220853/1-605   437  DSVALRANTALQTALPGAQINAQVSVTTKT...ANEIMLN....NSQ...............NKFLPKTVVIA..T...GVNNPE..NYK.......DD
WP_003687310/1-624   414  DSHADHYKTFFDAV.GKKE.KWSAT.MVSA.DACAYVEGYASRVFQNWA.ACRAVYRYAEEH.LPRYPKVVLAM.RWGS...QMPE.NS.RSLAYDA..G
YP_031545/1-343      341  ..........................L.........................................................................
WP_010975904/1-335   324  .................RVTAGLAGR..........................................................................
WP_011037591/1-364        ....................................................................................................
AAA86375/1-379            ....................................................................................................
AAA75102/1-357            ....................................................................................................
P08888/1-367              ....................................................................................................
Q52778/1-373         355  ..........................I.........................................................................
P23214/1-333         327  ..............SPK...................................................................................
WP_009895914/1-394        ....................................................................................................
WP_004545264/1-394        ....................................................................................................
NP_706267/1-390      378  ..........VKELIP....................................................................................
EFW62204/1-382       375  ..........IRGIIK....................................................................................
WP_000282635/1-349        ....................................................................................................
Q54131/1-367         358  ........................I.K.........................................................................
WP_004194788/1-412   392  .............WGPG...................................................................................
WP_001230914/1-332   329  ..........................I.........................................................................
WP_000170108/1-342   332  ........................V.L.........................................................................
P37669/1-331              ....................................................................................................
Q70J69/1-422              ....................................................................................................
```

WP_000400612/1-640	519	....TIKKIKEASPDSRIIFI.G....PV.......PEWNANLVKIISNYLSEFKKT.PPLYMTYGLNSE.ISE.WDSYFSNNVPKMGI.EYISAYKALC
WP_000639473/1-609	490	....TIKFLKDNK.VKNIIIV.G....P.....F..PVWKKTMIDTIEDMGINSGRT.VPWSMT.DETRN.LRD.NDKYLRELAKEHSLTYISPLETMC.
SIU02679/1-640	519	....TIKEIKKVSPQSRLIVV.G....PV.......PEWNANLVKVISNYTSEFKKT.PPIYMSYGLNDE.IKG.WDKFFEENVPKLGA.EYISAYSALC
AAX87447/1-622	494	FKARFKNTVKQLAMQKP.VYVFA....NN...SSVSRSPL..RGYLLENY..GLEKYLTP...I.HRMGD.IDA.SNKIIHDLVKDIPNVYWVDAQQYLP
CAC99369/1-622	541	....LNDLLDQFDKA.TIYLV.N...TRV...P...RGWQS................................D.VNKSIANAA.SRPNVTVVDWYSRSS
WP_002245844/1-622	495	FPARFRETVKRIAAVKP.VYVFA....NN...TSISRSPL..REEKLKRF..AANQYLRP...I.QAMGD.IGK.SNQAVFDLIKDIPNVHWVDAQKYLP
WP_011101182/1-660	572	....AEQVVKLIGKDRQIFWV.T...AHV...PT..QSWQN................................Q.VNAQIAKTAKKHANVHVIDWHGRAQ
YP_004888877/1-615	516	....TMKIIHDLEPGHKLILM.TPYNARA...D...ADWNS................................SKLAVLERRLPAKYKFITVADWGKIAA
WP_000379821/1-603	515	....LNELLDSFGKA.DIYLV.S...IRV...P...RDYEG................................R.INKLIYEAAEKRSNVHLVDWYKASA
WP_021723064/1-605	520	....IDAAMKVAGNK.PVYWI.N...VHA...D...RVWAK................................P.NNNLLKKMAKKYKNLKIIDWNKKAS
WP_001220853/1-605	500	....WDSIVKNLPKGHHMILV.TPYEGDKTKET...YAIVE.................................KAAAYMRELAEKTPYITIADWNQVAK
WP_003687310/1-624	500	FFQKFDRMLHKLSSEKQAVYLMA....DN...LA..SSYNVQRAYILSSRIPGCRQTLRP.......DDESTLK.ANARIRELAAKYPNVYIIDAAAYIP
YP_031545/1-343	342	....V...
WP_010975904/1-335		....
WP_011037591/1-364		....
AAA86375/1-379		....
AAA75102/1-357	338	.....................................EWL..KSKLLQ..
P08888/1-367	362	....R...........
Q52778/1-373	356	....VV.....................GAPPRPSLLKS........
P23214/1-333		....
WP_009895914/1-394	376	....................................ER...W........
WP_004545264/1-394	376	....................................DR...W........
NP_706267/1-390		....
EFW62204/1-382		....
WP_000282635/1-349		....
Q54131/1-367	360	....FK.........................RDFRI..........
WP_004194788/1-412	396	....................................QGWKY................................C.L........................
WP_001230914/1-332	330	....VK..
WP_000170108/1-342	334	....LP........................QKYIKL..........
P37669/1-331		....
Q70J69/1-422	398	....PVPVTSPQSS..

```
WP_000400612/1-640  599 NESGCLTRVGNGPDFI.TAVDWGHLTK......PGSDFLFNKIGNKI.....IK..........
WP_000639473/1-609  568 TESYCKAIIGNRIAYP.IQYDNAHLTP......EGSGWFIEEVKKQI.....S.......K...
SIU02679/1-640      599 NESGCLTRVGDGPDFV.TAVDWGHLTK......PGSDFLMKKIGHLI.....IR..........
AAX87447/1-622      576 KDS....VMAEG..KY.LYGDQDHLTN......FGAYYMAKEFSKYQRV...MTPEQV.KKLYE
CAC99369/1-622      592 GQ.........S..QY.FAPDGVHLTK......AGAQAYVAMLTSVM.....N........K..
WP_002245844/1-622  577 KNT....VEIYG..RY.LYGDQDHLTY......FGSYYMGREFHKHERL...LKSSRD.GAL.Q
WP_011101182/1-660  626 NQ.........S..GW.FADDNVHPNT......TGNRQLTNLIANRI.....AEVNN....N..
YP_004888877/1-615  573 QH.........P..EVFKGTDGVHFGG....IRAGDILYAKVINQAL.....TAAKQTPAKPA.
WP_000379821/1-603  567 GH.........P..EY.FAYDGIHLEY......AGSKALTDLIVKTM.....ETHATN..KK..
WP_021723064/1-605  572 GQ.........S..SW.FYSDNIHPKG......TGAEKYAALVANSL.....TDVE.....K..
WP_001220853/1-605  559 EH.........P..EIWAGTDQVHFGSESSTIEAGAKLYADTIATAL.....QTAQDKPVKSK.
WP_003687310/1-624  583 AD....FQIG...GLP.VYSDKDHINP......YGGTELAKRFSEKQRF...LDTRHN.H....
YP_031545/1-343     343 ............................................................P...
WP_010975904/1-335  333 ..............................................A.....A........E..
WP_011037591/1-364  354 ....................................PWT........LGARPVS......A...
AAA86375/1-379      354 ...................TQVRHFQN......HQSMWRVRVSHRQRRSA..........Q...
AAA75102/1-357      347 ........................................KNTSN..KL...AY.....ST...
P08888/1-367        363 .............................................RL.....SVA.........
Q52778/1-373        369 .................................QS...........V.....I.......N...
P23214/1-333        330 ......................LSLD......................................
WP_009895914/1-394  379 .................SRSRNVE..S......RG............SV....TSA.......Q
WP_004545264/1-394  379 .................AHARNVE..N......RK............SI....ASV.......P
NP_706267/1-390     384 ............................................TLT.....N.NN......Q.
EFW62204/1-382      381 ............................................N.................T.
WP_000282635/1-349      ................................................................
Q54131/1-367        367 ............................................................N...
WP_004194788/1-412  403 ....................ARLTMKK......EGV............................
WP_001230914/1-332  332 ............................................................L...
WP_000170108/1-342  342 ............................................................K...
P37669/1-331        325 ....................DR......................NRL.....VS..........
Q70J69/1-422        408 ....APPA...............H..Q......AGAAH.......MV......ER.........
```
