# Supplementary material for: Acetylation of Surface Carbohydrates in Bacterial Pathogens Requires Coordinated Action of a Two-Domain Membrane-Bound Acyltransferase
Source: mBio. 2020 Aug 25;11(4):e01364-20. doi: 10.1128/mBio.01364-20 (PMC7448272; doi:10.1128/mBio.01364-20)
Supplement: FIG S2 [file mBio.01364-20-sf002.pdf]

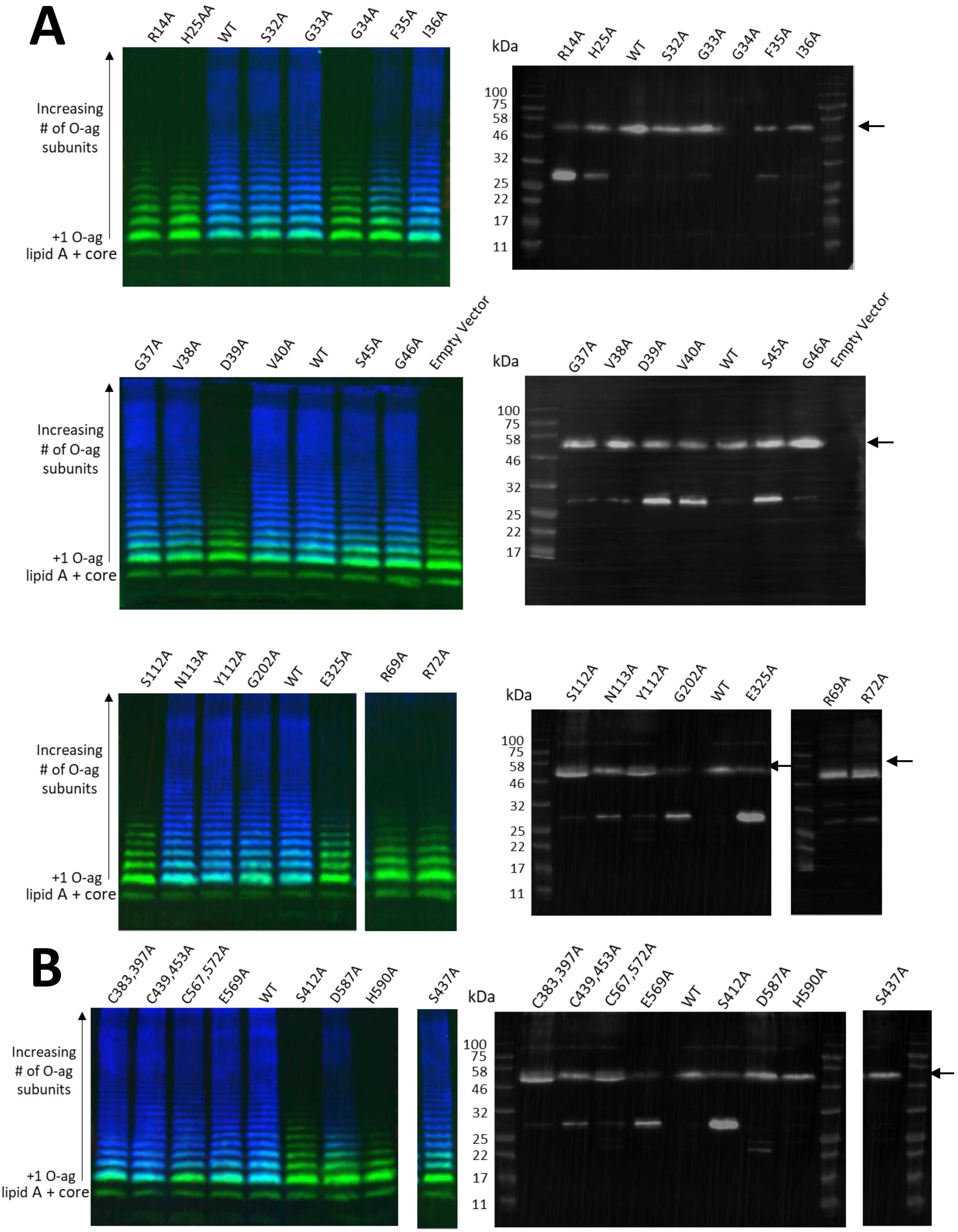

Fig. S2. Functional analysis of OafA point mutants *in situ*

Left panel shows LPS western blot with crude LPS extracts from *Salmonella* ser. Typhimurium basal O-antigen strain expressing OafA point mutant variants in (A) the membrane domain and (B) the periplasmic domain. O:5 antibody binding (Blue) shows abequose acetylation and *Salmonella* LPS core antibody binding (Green) acts as a loading control. Right panel shows corresponding anti-His western blot for expression of His tagged OafA. Arrow indicates full length OafA protein.
