# Supplementary material for: Acetylation of Surface Carbohydrates in Bacterial Pathogens Requires Coordinated Action of a Two-Domain Membrane-Bound Acyltransferase
Source: mBio. 2020 Aug 25;11(4):e01364-20. doi: 10.1128/mBio.01364-20 (PMC7448272; doi:10.1128/mBio.01364-20)
Supplement: TABLE S2 [file mBio.01364-20-st002.pdf]

**Table S2.** Molecular biology materials. Bacterial strains and primers used in this study. Primers for cloning of OafA and OafB constructs and creation of OafA point mutant variants on the pBADcLIC_WT-OafA plasmid. Amp = Ampicillin 100 µg/ml, Kan = Kanamycin 50 µg/ml.

| **Strain Name** | **Strain Number** | **Genotype** | **Plasmids** | **Source** | **Antibiotic** |
|---|---|---|---|---|---|
| Escherichia coli Strains | | | | | |
| XL1 Blue | | | | Agilent | |
| Origami2 | | *Δ(ara-leu)7697 ΔlacX74 ΔphoA Pvu*II *phoR araD139 ahpC galE galK rpsL* F′[*lac*$^{+}$ *lacI*$^{q}$ *pro*] (DE3) *gor522::*Tn*10 trxB* (Str$^{R}$, Tet$^{R}$) | | Novagen | |
| Origami2 + pETFPP_2-OafA$_{STM}^{C\text{-long}}$ | - | Origami2 | JP327 | This study | Kan |
| Origami2 + pETFPP_2-OafB$_{STM}^{C\text{-long}}$ | - | Origami2 | JP331 | This study | Kan |
| Origami2 + pETFPP_2-OafA$_{STM}^{C\text{-short}}$ | - | Origami2 | JP329 | This study | Kan |
| Origami2 + pETFPP_2-OafB$_{STM}^{C\text{-short}}$ | - | Origami2 | JP333 | This study | Kan |
| Salmonella ser. Typhimurium Strains | | | | | |
| LT2 | 346 | STM LT2 (ATCC strain number 19585, Lot number 215096) | - | ATCC | |
| LT2 Basal O-ag | 293 | STM LT2 ΔGtr all, ΔOafA | - | Davies *et al*. 2013 | |
| LT2 Basal O-ag + pBADcLIC | 993 | STM LT2 ΔGtr all, ΔOafA | 432 | This study | Amp |
| LT2 Basal O-ag + pBADcLIC_WT-OafA | 932 | STM LT2 ΔGtr all, ΔOafA | 433 | This study | Amp |
| LT2 Basal O-ag + pBADcLIC_R14A-OafA | 1023 | STM LT2 ΔGtr all, ΔOafA | 509 | This study | Amp |
| LT2 Basal O-ag + pBADcLIC_H25A-OafA | 1022 | STM LT2 ΔGtr all, ΔOafA | 508 | This study | Amp |
| LT2 Basal O-ag + pBADcLIC_S32A-OafA | 1009 | STM LT2 ΔGtr all, ΔOafA | 501 | This study | Amp |
| LT2 Basal O-ag + pBADcLIC_G33A-OafA | 1024 | STM LT2 ΔGtr all, ΔOafA | 510 | This study | Amp |
| LT2 Basal O-ag + pBADcLIC_G34A-OafA | 1014 | STM LT2 ΔGtr all, ΔOafA | 506 | This study | Amp |
| LT2 Basal O-ag + pBADcLIC_F35A-OafA | 1025 | STM LT2 ΔGtr all, ΔOafA | 511 | This study | Amp |

| | | | | | |
|---|---|---|---|---|---|
| LT2 Basal O-ag + pBADcLIC_I36A-OafA | 1026 | STM LT2 ΔGtr all, ΔOafA | 512 | This study | Amp |
| LT2 Basal O-ag + pBADcLIC_G37A-OafA | 1010 | STM LT2 ΔGtr all, ΔOafA | 502 | This study | Amp |
| LT2 Basal O-ag + pBADcLIC_V38A-OafA | 1011 | STM LT2 ΔGtr all, ΔOafA | 503 | This study | Amp |
| LT2 Basal O-ag + pBADcLIC_D39A-OafA | 1012 | STM LT2 ΔGtr all, ΔOafA | 504 | This study | Amp |
| LT2 Basal O-ag + pBADcLIC_V40A-OafA | 1015 | STM LT2 ΔGtr all, ΔOafA | 507 | This study | Amp |
| LT2 Basal O-ag + pBADcLIC_S45A-OafA | 1035 | STM LT2 ΔGtr all, ΔOafA | 521 | This study | Amp |
| LT2 Basal O-ag + pBADcLIC_G46A-OafA | 1013 | STM LT2 ΔGtr all, ΔOafA | 505 | This study | Amp |
| LT2 Basal O-ag + pBADcLIC_R69A-OafA | 1085 | STM LT2 ΔGtr all, ΔOafA | 540 | This study | Amp |
| LT2 Basal O-ag + pBADcLIC_R72A-OafA | 1086 | STM LT2 ΔGtr all, ΔOafA | 541 | This study | Amp |
| LT2 Basal O-ag + pBADcLIC_S112A-OafA | 1036 | STM LT2 ΔGtr all, ΔOafA | 522 | This study | Amp |
| LT2 Basal O-ag + pBADcLIC_N113A-OafA | 1078 | STM LT2 ΔGtr all, ΔOafA | 539 | This study | Amp |
| LT2 Basal O-ag + pBADcLIC_Y122A-OafA | 1037 | STM LT2 ΔGtr all, ΔOafA | 523 | This study | Amp |
| LT2 Basal O-ag + pBADcLIC_G202A-OafA | 1027 | STM LT2 ΔGtr all, ΔOafA | 513 | This study | Amp |
| LT2 Basal O-ag + pBADcLIC_E325A-OafA | 1028 | STM LT2 ΔGtr all, ΔOafA | 514 | This study | Amp |
| LT2 Basal O-ag + pBADcLIC_R379A-OafA | 1033 | STM LT2 ΔGtr all, ΔOafA | 519 | This study | Amp |
| LT2 Basal O-ag + pBADcLIC_C383,397A-OafA | 1001 | STM LT2 ΔGtr all, ΔOafA | 497 | This study | Amp |
| LT2 Basal O-ag + pBADcLIC_C439,453A-OafA | 1007 | STM LT2 ΔGtr all, ΔOafA | 499 | This study | Amp |
| LT2 Basal O-ag + pBADcLIC_C567,572A-OafA | 1002 | STM LT2 ΔGtr all, ΔOafA | 498 | This study | Amp |
| LT2 Basal O-ag + pBADcLIC_S437A-OafA | 1032 | STM LT2 ΔGtr all, ΔOafA | 518 | This study | Amp |
| LT2 Basal O-ag + pBADcLIC_E569A-OafA | 1030 | STM LT2 ΔGtr all, ΔOafA | 516 | This study | Amp |
| LT2 Basal O-ag + pBADcLIC_S412A-OafA | 933 | STM LT2 ΔGtr all, ΔOafA | 452 | This study | Amp |
| LT2 Basal O-ag + pBADcLIC_D587A-OafA | 934 | STM LT2 ΔGtr all, ΔOafA | 453 | This study | Amp |

| | | | | | |
|---|---|---|---|---|---|
| LT2 Basal O-ag + pBADcLIC_H590A-OafA | 1055 | STM LT2 ΔGtr all, ΔOafA | 527 | This study | Amp |

| OafA Mutants | Forward Primer | | Reverse Primer | |
|---|---|---|---|---|
| | Primer code | Sequence 5'-3' | Primer code | Sequence 5'-3' |
| R14A | oMV1403 | AAT GGG CTA GCG GCT TTT G | oMV1404 | TAT ATC GAG TCT GAA TTT CTT GTA GAT CAT AGC |
| H25A | oMV1407 | GTG CTG TAT GCG TTC GGT GT | oMV1408 | AAC ACT TAT CAA TGC AAA AGC TCT TAG CC |
| S32A | oMV1399 | CT TAT GTG GCG GGT GG | oMV1400 | GTACACCGAAGTGATACAGCA |
| G33A | oMV1409 | CT TAT GTG TCA GCG GGC TTT | oMV1410 | G TAC ACC GAA GTG ATA CAG CAC |
| G34A | oMV1382 | GTG TCA GGT GCG TTT ATA GGT | oMV1383 | ATA AGG TAC ACC GAA GTG ATA CAG |
| F35A | oMV1411 | G TCA GGT GGC GCG ATA GG TG | oMV1412 | ACA TAA GGT ACA CCG AAG TGA TAC AGC AC |
| I36A | oMV1413 | GGT GGC TTT GCG GGT GTA G | oMV1414 | TGA CAC ATA AGG TAC ACC GAA GTG ATA CAG |
| G37A | oMV1388 | GGC TTT ATA GCG GTA GAT GTT TTC | oMV1389 | ACC TGA CAC ATA AGG TAC ACC |
| V38A | oMV1390 | C TTT ATA GGT GCG GAT GTT TTC TTT | oMV1391 | CCACCTGACACATAAGGTACA |
| D39A | oMV1392 | C TTT ATA GGT GTA GCG GTT TTC TTT GTA | oMV1391 | CCACCTGACACATAAGGTACA |
| V40A | oMV1393 | GGT GTA GAT GCG TTC TTT GTA ATT TCT | oMV1394 | TAT AAA GCC ACC TGA CAC ATA AGG TA |
| S45A | omv1505 | CTTTGTAATTGCGGGTTTTCTTATGAC | oMV1506 | AAAACATCTACACCTATAAAGCCACCT |
| G46A | oMV1395 | GTA ATT TCT GCG TTT CTT ATG ACT G | oMV1396 | AAA GAA AAC ATC TAC ACC TAT AAA GCC |
| R69A | oMV1553 | GAT TTT TAT ATT GCA GCG TTC CTA AG | oMV1554 | AAG TAC TCC TTT GTG GTC TAC |
| R72A | oMV1555 | TAT TGC AAG ATT CCT AGC GAT TGT A | oMV1556 | TAA AAA TCA AGT ACT CCT TTG TGG TC |
| S112A | oMV1507 | GTCTTTACTTTTTTATGCGAATAATTA | oMV1508 | GATATTGCGTTCTTGCTAAGTG |
| N113A | oMV1533 | ACTTTTTTATTCAGCGAATTATTACG | oMV1534 | AAGACGATATTGCGTTCTTGC |
| Y122A | oMV1509 | CACTCTAGTGCGTTCGACTCA | oMV1510 | AATTGCGTAATAATTATTTGAATAAAAAAGTA |
| G202A | oMV1415 | GAA ATG CTG GCT GCG GGC | oMV1416 | CCA TGC CCT GGT AGG GAT AAG ATA GA |
| E325A | oMV1475 | C AGA ACA ATT GCG AAC ACG CT | oMV1476 | TATGAAATATCCCCAAGCGCAAAAG |

| | | | | |
|---|---|---|---|---|
| C383S | oMV1332 | AGG CCT GAT ATT AGC TTC CTC AAT CCA | oMV1333 | CCA GGG AGA GTT GTC CAT ACG ATA |
| C397S | oMV1351 | GCA TTC TCA AAA AGC CAG GAT AAA TAG | oMV1352 | TGA ATA ATC TTG ATC TGG ATT GAG GAA |
| C439S | oMV1336 | GCA AGC TTG AGC CAA CCA AT | oMV1337 | AGT TCT CTG CGT AAT GTT AAG TGA ATT TCC |
| C453S | oMV1334 | GAC AGG CCG TAT AGC AAA GAC ATC AAT | oMV1378 | ATC TTT TTG AAG CCC AAT GAT TGG TGG |
| C567S | oMV1353 | CTT GAG ACT ATG AGC ACA GAA AGT TAT | oMV1366 | TGG TGA AAT ATA AGT AAG GGA ATG CTC |
| C572S | oMV1355 | GT ACA GAA AGT TAT AGC AAA GCA ATA ATA | oMV1356 | CAT AGT CTC AAG TGG TGA AAT ATA AG |
| S437A | oMV1491 | CAGAGAACTGCAGCGTTGTGC | oMV1492 | CGTAATGTTAAGTGAATTTCCAAATACCGATTTC |
| E569A | oMV1473 | CT ATG TGT ACA GCG AGT TAT TGC | oMV1474 | TCT CAA GTG GTG AAA TAT AAG TAA GGG |
| S412A | oMV1152 | ATG GGG TGA CGC GCA TGC CGC | oMV1153 | ACA ACA AAA GAC TTT TCA GTC ATT TTA TCC TGA CAT TTT G |
| D587A | oMV1150 | ATA CAA TAT GCC AAT GCG CAC C | oMV1151 | AGG GTA AGC AAT TCT ATT CC |
| H590A | oMV1148 | TGA CAA TGC GGC CCT AAC ACC AGA AG | oMV1149 | TAT TGT ATA GGG TAA GCA ATT C |
| *OafA$_{SPA}^{long}$ | - | TCC AGG GAC CAG CAA TGA AGC AAG TCG TTG AGT ATC G | - | TGA GGA GAA GGC GCG TCA TTA TTT TGA AAT CTG CTT TTT CAC |
| *OafB$_{SPA}^{long}$ | - | TCC AGG GAC CAG CAA TGA AAA GCG CCG GTG AAT AC | - | TGA GGA GAA GGC GCG TCA TTT AAT GAT TTT ATT GCC AAT CTT G |
| *OafA$_{SPA}^{short}$ | - | TCC AGG GAC CAG CAA TGA GGC CTG ATA TTT GC | - | TGA GGA GAA GGC GCG TCA TTA TTT TGA AAT CTG CTT TTT CAC |
| *OafB$_{SPA}^{short}$ | - | TCC AGG GAC CAG CAA TGC GCG GCG GCA TTT GC | - | TGA GGA GAA GGC GCG TCA TTT AAT GAT TTT ATT GCC AAT CTT G |
