# Supplementary material for: Acetylation of Surface Carbohydrates in Bacterial Pathogens Requires Coordinated Action of a Two-Domain Membrane-Bound Acyltransferase
Source: mBio. 2020 Aug 25;11(4):e01364-20. doi: 10.1128/mBio.01364-20 (PMC7448272; doi:10.1128/mBio.01364-20)
Supplement: TABLE S3 [file mBio.01364-20-st003.pdf]

Table S3. X-ray crystallography data and statistics for the structure of $OafB_{SPA}^{C\text{-long}}$. Values in parenthesis correspond to the highest resolution shell unless otherwise stated.

**Data collection**	
Beamline	I04-1
Space group	$P2_12_12_1$
Cell dimensions	
a, b, c (Å)	56.74, 58.43, 90.03
α, β, γ (°)	90, 90, 90
Wavelength (Å)	0.9282
Resolution (Å)	47.39 – 1.08 (1.10 – 1.08)
No. unique reflections	126378 (6232)
Completeness (%)	100 (99.7)
Multiplicity	7.7 (6.4)
I/σ(I)	12.2 (1.2)
R_{merge} (%)	7.2 (102.5)
R_{pim} (%)	2.8 (52.9)
$CC_{1/2}$	0.999 (0.651)
**Refinement**	
Resolution	47.39 – 1.08
No. reflections (test set)	119910 (6379)
R_{work} / R_{free} (%)	13.6 / 14.9
CC_{work}	0.981
CC_{free}	0.979
No. atoms:	
Protein	2096
Water	360
Ligand	19
B factors ($Å^2$):	
Protein	13.49
Water	29.51
Ligand	19.55
RMSD bond lengths (Å)	0.0060
RMSD bond angles (°)	1.451
Ramachandran plot (%)	
Favoured	99
Allowed	1
Outliers	0
**Data deposition**	
PDB ID	6SE1
