# Supplementary material for: Acetylation of Surface Carbohydrates in Bacterial Pathogens Requires Coordinated Action of a Two-Domain Membrane-Bound Acyltransferase
Source: mBio. 2020 Aug 25;11(4):e01364-20. doi: 10.1128/mBio.01364-20 (PMC7448272; doi:10.1128/mBio.01364-20)
Supplement: FIG S3 [file mBio.01364-20-sf003.pdf]

Fig. S3. Structure based sequence alignment of OafB, OafA and closest structural homologues. Residues conserved in >50% highlighted blue, catalytic and oxyanion hole residues are indicated by an arrow. Abbreviations and details of sequences used in methods section.

```
OafB-S.PA    334  .EKPLRNSRSKYIT......AILL.TLSVFGTGLIGAFIFHINGVKDR.......  373
OafB-S.Tym   334  .EKPLRHSRKKSIT......TIIL.AVVVFGSGIFGIVTYSMNGIKER.......  373
OafA-S.Tym   325  .ENTLRKRVK..........LQFN.IVLFSSTLALCLFVMFTKGVSFR.......  360
OafA-H.inf   345  .EQPIRKS...........KLNFK.QSFLYIYFIPSLLLLGF.............  373
OatA-S.pne   347  ..EPWIAGKNTPIVQTLRPLPYIH.AILAAGTGILTIIVCTVTLLAPQ.......  391
OatA-S.aur   357  .ETPIRKKGFKAFAFLPKKKGQFARTVLVILLLVPSIVVLSGQFDALGKQHEAEK  410
Ape1-N.men     1  GSLPVASVSLDTVTV..SPSAPYT................DTNGLLTD.......   30
TAP1-E.col     1  ...................MMNFN.NVFRWHLPFLFLVLLT..............   21
RGAE-A.acu     1  .......................................................   -1
5B5S-T.cel     1  .......................................................   -1
2VPT-C.the     1  .......................................................   -1


OafB-S.PA    374  ......EINKSAGEYASVTDVY.NYYKYGELLRGGICHSVQ..LTAAISNGCIKN  419
OafB-S.Tym   374  ......SVNKSAGEYASVTNVY.DYYKYGELLRGGICHSVL..LKDAISNGCIKN  419
OafA-S.Tym   361  ......FSDTLKQ.VVEYRMDN.......SPWRPDICFLNPDQDYSAFSKCQDKM  401
OafA-H.inf   374  .......NLYKRQ.TIRAEKEHIEQSI.....................PVSNENH  399
OatA-S.pne   392  ......VGAFETDLTVNGLKQAATNIGQTK.............VMAERADANSLG  427
OatA-S.aur   411  KEKKTEFKTTKKK.VVKKDKQEDKQTA......................NSKEDI  442
Ape1-N.men    31  ......YGNASASPWMKKLQSVAQ............................GSG   51
TAP1-E.col    22  ...................................................FRAA   25
RGAE-A.acu     1  .......................................................   -1
5B5S-T.cel     1  .......................................................   -1
2VPT-C.the     1  ......................................................K    1

                            ↓
OafB-S.PA    420  GKHNIFIIGDSYAAA.........LFNGLSH.YIDNKGSD...............  449
OafB-S.Tym   420  SRNNIFIIGDSYAAA.........LYNGLSS.YIKNNNEK...............  449
OafA-S.Tym   402  TEKSFVVWGDSHAAH.........LMPGLKSVFGNS...................  428
OafA-H.inf   400  YPAKVIILGDSHSSH.........LEAFLNYVG.NKEGWK...............  429
OatA-S.pne   428  IADGTMLIGDSVALRANT......ALQTAL.........................  451
OatA-S.aur   443  KKSSPLLIGDSVMVD.........IGNVFTKKI......................  466
Ape1-N.men    52  ETFRILQIGDSHTAGDF.......FTDSLRKRLQKTWGDGGIGWVYPANVKGQRM   99
TAP1-E.col    26  AADTLLILGDSLSAGYRMSA.SAAWPALLNDKW....QSK...............   60
RGAE-A.acu     1  ..TTVYLAGDSTMAKNGGGSGTNGWGEYLASYLS.....................   32
5B5S-T.cel     1  .MVKVMLLGDSITEITC.......WRPLVW.EQITSAGLAG..............   32
2VPT-C.the     2  TIIKIMPVGDSCTEGMGGGE.MGSYRTELYRRLLTQAGLS...............   40


OafB-S.PA    450  ........YIIS...........................................  453
OafB-S.Tym   450  ........YVIS...........................................  453
OafA-S.Tym   429  ........LNIT...........................................  432
OafA-H.inf   430  ........ADIF...........................................  433
OatA-S.pne   452  ........PGAQ...........................................  455
OatA-S.aur   467  ........PNAQ...........................................  470
Ape1-N.men   100  AAVRHNGNWQSLTSRNNTGDFPLGGILAHTGSGGSMTLTASDGIASKQRVSLFAK  154
TAP1-E.col    61  ........TSVV...........................................   64
RGAE-A.acu    33  ........ATV............................................   35
5B5S-T.cel    33  .......SVDFVG.SMNDLQPNCSRP.............................   50
2VPT-C.the    41  ........IDFVGSSQRRSGP.SSS..............................   56
```

```
OafB-S.PA     454  .......................................................  453
OafB-S.Tym    454  .......................................................  453
OafA-S.Tym    433  .......................................................  432
OafA-H.inf    434  .......................................................  433
OatA-S.pne    456  .......................................................  455
OatA-S.aur    471  .......................................................  470
Ape1-N.men    155  PLLAEQTLTVNGNTVSANGGGWQVLDTGAALPLTIHTEMPWDIGFINIENPAGGI  209
TAP1-E.col     65  .......................................................   64
RGAE-A.acu     36  .......................................................   35
5B5S-T.cel     51  ....................................................QGF   53
2VPT-C.the     57  .....................................................LP   58

                                ↓
OafB-S.PA     454  ...QMTD.....GNAPPLFVDGKDDLQRSVITLNN...NRINEIKRV..QPEVVL  495
OafB-S.Tym    454  ...QMTD.....GNAPPLFVSGKDDLQRDVGSINA...DRIKEIGIV..KPEIVL  495
OafA-S.Tym    433  ...QRTASLCPPIIGLQKDDRPYC......KDIND...MVAKEISDN..KPTTVL  473
OafA-H.inf    434  ...KDKFECSFIVN.EQYQLDPNC......QSVWQ...KDSQYKAIF..ISAFYD  473
OatA-S.pne    456  ...INAQ.....VS.......VTT......KTANE...IMLNNSQNKF.LPKTVV  485
OatA-S.aur    471  ...IDGK.....VG.......RQL......VDATP...IVKSQYKDYAKKGQKVV  501
Ape1-N.men    210  TVSAMGI.....NG.......AQL......TQWSKWRADRMNDLAQT..GADLVI  244
TAP1-E.col     65  ...NASI.....SG.......DTS......QQGLA...RLPALLKQH..QPRWVL   93
RGAE-A.acu     36  ..VNDAV.....AG.......RSA......RSYTR..EGRFENIADVVTAGDYVI   68
5B5S-T.cel     54  DPDHEGH.....SG.......WQA......YDIAR..NNIAGWVQNT..KPDIVQ   86
2VPT-C.the     59  DKDHEGH.....SG.......WTI......PQIAS...NINNWLNTH..NPDVVF   90

                             ↓
OafB-S.PA     496  LTWSV..RGTNG...VHDKK...................LAI....DALSLTIKK  522
OafB-S.Tym    496  LTWSV..RGSNG...VHDKK...................LAI....EALSLTIKE  522
OafA-S.Tym    474  MSALWPVY...............................PMR....DYLPETIKF  493
OafA-H.inf    474  LRMGGQPVPRFRPETFIEP....................DFK....ARFKNTVKQ  504
OatA-S.pne    486  IATGVNNPE..............................NYK....DDWDSIVKN  506
OatA-S.aur    502  VELGTNGAFTK...................................DQLNELLDS  521
Ape1-N.men    245  LSYGTNEAFNNNID.......................IADTE....QKWLDTVRQ  272
TAP1-E.col     94  VELGGNDGLRG....FQPQ....................QTE....QTLRQILQD  120
RGAE-A.acu     69  VEFGHNDGGSLS...TDNGRTDCSGTGAEVCYSVYDGVNETILTFPAYLENAAKL  120
5B5S-T.cel     87  FMLGTNDVNIGH...RNAD....................SII....GSYTIMLNA  114
2VPT-C.the     91  LWIGGNDLLLNG.........................NLLNA....TGLSNLIDQ  116

OafB-S.PA     523  IKEASPDSRIIFIGPVPE...........WNANLVKIISNYLSEFKKTPPLYMTY  566
OafB-S.Tym    523  IKKVSPQSRLIVVGPVPE...........WNANLVKVISNYTSEFKKTPPIYMSY  566
OafA-S.Tym    494  LKDNKV.KNIIIVGPFPVW...........KKTMIDTIEDMGINSGRTVPWSM.T  535
OafA-H.inf    505  LAMQK...PVYVFANNSSVSR.......SPL...RGYLLENYGLEKYLTPI....  542
OatA-S.pne    507  LP...KGHHMILVTP.YEG..........DKT......................K  525
OatA-S.aur    522  FGK....ADIYLVSIRVPR....................................  536
Ape1-N.men    273  IRDSLPAAGILIIGAPESLKNTLGVCGTRPV........................  303
TAP1-E.col    121  VKAAN..AEPLLMQIRLPANY.......GRR........................  142
RGAE-A.acu    121  FTAKG..AKVILSSQTPNNPW.......ETGTFVN....................  146
5B5S-T.cel    115  MRAANPRVKVIVDKIIPTSWS..................................  135
2VPT-C.the    117  IFTVKPNVTLFVADYYPWPE...................................  136
```

```
OafB-S.PA    567  GLNSEISEWDSYFSNNVPKMG.....IEYISAY.KALCNESGC..LTRVGNGPDF  613
OafB-S.Tym   567  GLNDEIKGWDKFFEENVPKLG.....AEYISAY.SALCNESGC..LTRVGDGPDF  613
OafA-S.Tym   536  DETRNLRDNDKYLRELAKEH.....SLTYISPL.ETMCT.ESYCKAIIGNRIAYP  583
OafA-H.inf   543  HRMGDIDASNKIIHDLVKDIP....NVYWVDAQ.QYLPKDS.......VMAEGKY  585
OatA-S.pne   526  ETYAIVEKAAAYMRELAE.KTP...YITIADWN.QVAKE............HPEI  563
OatA-S.aur   536  ...DYEGRINKLIYEAAEKRS....NVHLVDWY.KASAG............HPEY  571
Ape1-N.men   304  ....RLTEVQQMQRRVARQG.....QTMFWSWQ..NAMGGICSMK...NWLNQGW  344
TAP1-E.col   143  ....YNEAFSAIYPKLAKEFD.....VPLLPFFMEEVYL............KPQW  176
RGAE-A.acu   147  ....SPTRFVEYAELAAEVA.....GVEYVDHW..SYVDSIYETL..GNATVNSY  188
5B5S-T.cel   136  ..DATIEAVNTAIPGWVQQQTTAESPVVIADCS.RAAGF............TNDM  175
2VPT-C.the   137  ....AIKQYNAVIPGIVQQKANAGKKVYFVKLS..EIQF...........DRNTD  174

OafB-S.PA    614  ITAVDWGHL......TKPGSDFLFNKIGNK........IIK..............  640
OafB-S.Tym   614  VTAVDWGHL......TKPGSDFLMKKIGHL........IIR..............  640
OafA-S.Tym   584  I.QYDNAHL......TPEGSGWFIEEVKKQ........ISK..............  609
OafA-H.inf   586  L.YGDQDHL......TNFGAYYMAKEFSKYQRVMTPEQVKKL.......YE....  622
OatA-S.pne   564  WAGTDQVHFGSESSTIEAGAKLYADTIATA........LQTA.......QDKPVK  603
OatA-S.aur   572  F.AYDGIHL......EYAGSKALTDLIVKT........METH.......ATNKK.  603
Ape1-N.men   345  A.AKDGVHF......SAKGYRRAAEMLADS........LEEL.......VRSAAI  377
TAP1-E.col   177  M.QDDGIHP......NRDAQPFIADWMAKQ........LQPL.......VNHDS.  208
RGAE-A.acu   189  F.PIDHTHT......SPAGAEVVAEAFLKA........VVCTGTSLKSVLTTTSF  228
5B5S-T.cel   176  L.RDDGVHP......NSKGDQFIAGQIGPK........LIQL.......IKDVS.  207
2VPT-C.the   175  I.SWDGLHL......SEIGYKKIANIWYKY........TIDI.......LRRALA  207

OafB-S.PA    641  .....  640
OafB-S.Tym   641  .....  640
OafA-S.Tym   610  .....  609
OafA-H.inf   623  .....  622
OatA-S.pne   604  SK...  605
OatA-S.aur   604  .....  603
Ape1-N.men   378  R....  378
TAP1-E.col   209  .....  208
RGAE-A.acu   229  EGTCL  233
5B5S-T.cel   208  .....  207
2VPT-C.the   208  G....  208
```
