# Supplementary material for: Acetylation of Surface Carbohydrates in Bacterial Pathogens Requires Coordinated Action of a Two-Domain Membrane-Bound Acyltransferase
Source: mBio. 2020 Aug 25;11(4):e01364-20. doi: 10.1128/mBio.01364-20 (PMC7448272; doi:10.1128/mBio.01364-20)
Supplement: FIG S4 [file mBio.01364-20-sf004.pdf]

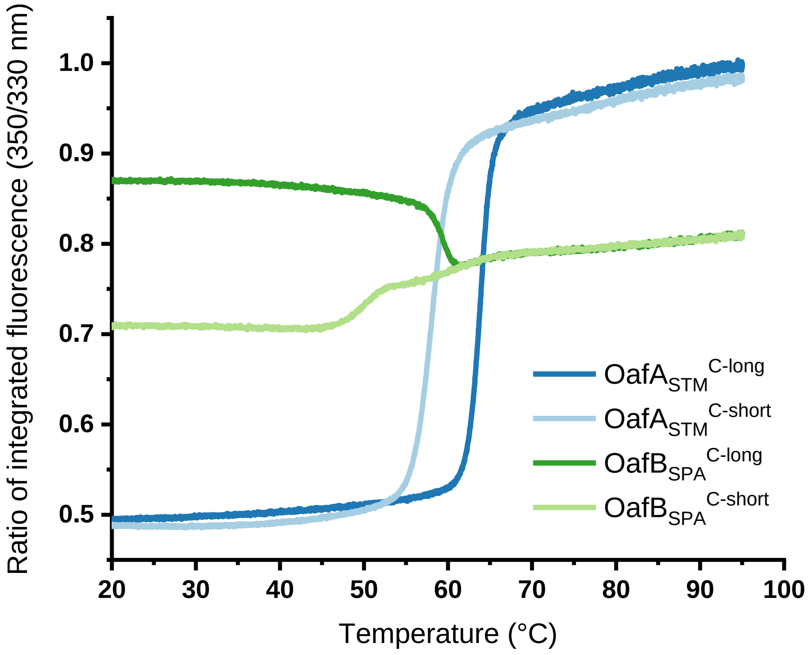

Fig. S4. Melting curves of OafA$_{STM}^{C\text{-}long}$, OafA$_{STM}^{C\text{-}short}$, OafB$_{SPA}^{C\text{-}long}$, OafB$_{SPA}^{C\text{-}short}$, with melting temperatures of OafA$_{STM}^{C\text{-}long}$ = 63.8 ^{o}C, OafA$_{STM}^{C\text{-}short}$ = 58.1 ^{o}C, OafB$_{SPA}^{C\text{-}long}$ = 58.9 , OafB$_{SPA}^{C\text{-}short}$ = 50.0 ^{o}C.
