# Supplementary material for: Acetylation of Surface Carbohydrates in Bacterial Pathogens Requires Coordinated Action of a Two-Domain Membrane-Bound Acyltransferase
Source: mBio. 2020 Aug 25;11(4):e01364-20. doi: 10.1128/mBio.01364-20 (PMC7448272; doi:10.1128/mBio.01364-20)
Supplement: FIG S5 [file mBio.01364-20-sf005.pdf]

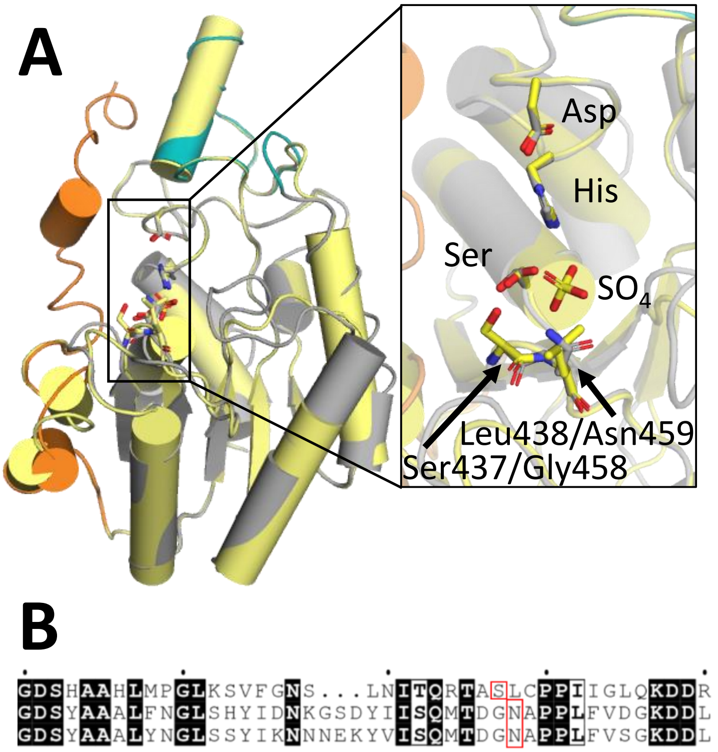

Fig. S5. Comparison of potential oxyanion hole residues in OafA and OafB. A. Homology model of $OafA_{STM}^{C\text{-}long}$ (yellow) modelled on the structure of OafB (grey, extension in orange and additional helix in teal). Catalytic triad and potential oxyanion hole residues shown as sticks. Residues indicated with OafA first. Both Ser 437 side chain and Leu 438 backbone amide are in close proximity to catalytic triad and active site sulfate. B. Sequence alignment of OafA from Salmonella ser. Tyhimurium and OafB from Typhimurium and Paratyphi A serovars of Salmonella from OafA residues 410-450. Alignments were carried out using Tcoffee with default settings. Red Box highlights predicted replacements for catalytic block II glycine.
