# Supplementary material for: Acetylation of Surface Carbohydrates in Bacterial Pathogens Requires Coordinated Action of a Two-Domain Membrane-Bound Acyltransferase
Source: mBio. 2020 Aug 25;11(4):e01364-20. doi: 10.1128/mBio.01364-20 (PMC7448272; doi:10.1128/mBio.01364-20)
Supplement: FIG S6 [file mBio.01364-20-sf006.pdf]

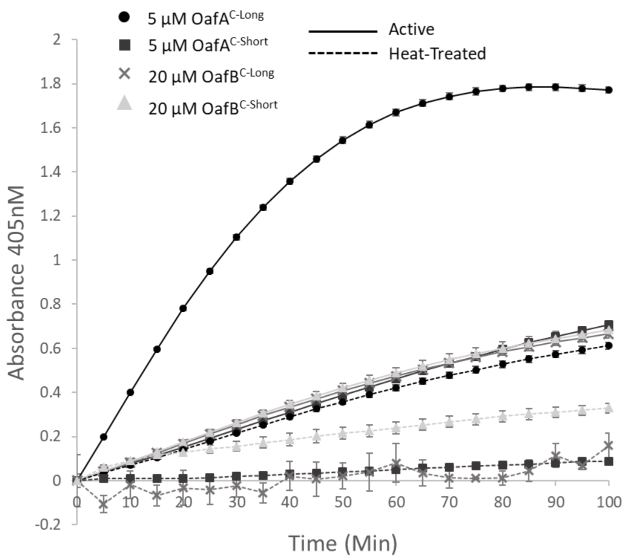

Figure S6| *In vitro* acetyl-esterase activity of C-terminal OafA and OafB assessed by hydrolysis of pNitrophenyl acetate (pNPA). Solid line = Active protein, dashed line = Heat treated protein. Error bars = SEM, N=3. Some error bars are obscured by point markers. 'C-Long' constructs comprise the SGNH domain with full $SGNH_{ext,}$ 'C-Short' constructs comprise the SGNH domain with fewer $SGNH_{ext}$ residues to expose the SGNH domain active site. See Figure 1 for details of the C-terminal OafA and OafB constructs.
