# Supplementary material for: Acetylation of Surface Carbohydrates in Bacterial Pathogens Requires Coordinated Action of a Two-Domain Membrane-Bound Acyltransferase
Source: mBio. 2020 Aug 25;11(4):e01364-20. doi: 10.1128/mBio.01364-20 (PMC7448272; doi:10.1128/mBio.01364-20)
Supplement: FIG S7 [file mBio.01364-20-sf007.pdf]

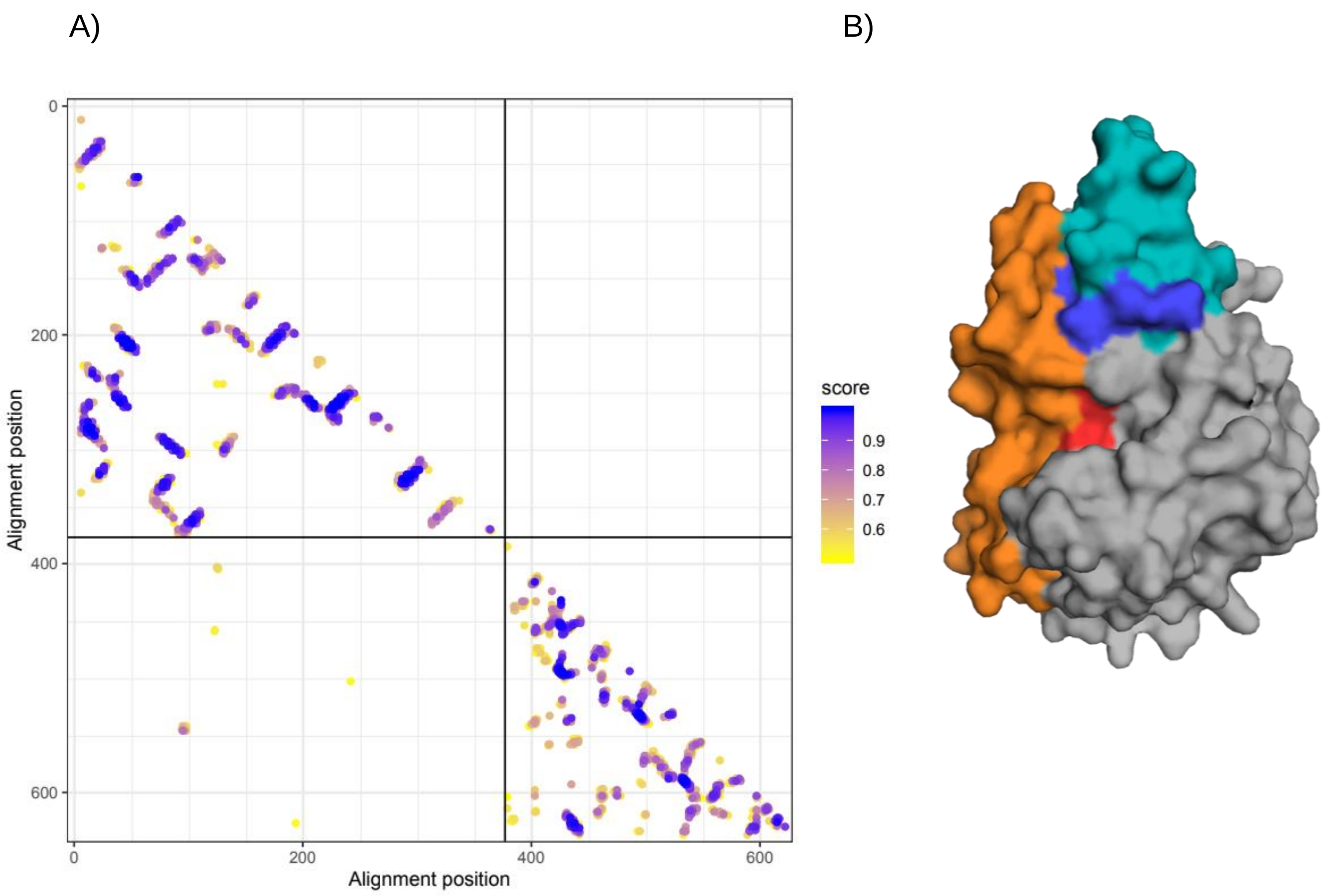

Fig. 7. A) Predicted contact map for OafB based on a correlated mutation analysis using the RaptorX webserver. The horizontal/vertical line marks residue 377, which forms the boundary at the end of the AT3 domain. High confidence interactions within the AT3 domain (top left) and the SGNH domain (bottom right), while a single high scoring interaction between the AT3 (93-97) and SGNH (524-546) is marked (bottom left). B) Structure of $OafB_{SPA}^{C\text{-}long}$ with residues (542-546) predicted to interact with the acyltransferase domain coloured blue. The extension is coloured orange, the additional helix coloured teal, and catalytic triad coloured red.
